# Association of Rare Coding Mutations With Alzheimer Disease and Other Dementias Among Adults of European Ancestry

**DOI:** 10.1001/jamanetworkopen.2019.1350

**Published:** 2019-03-29

**Authors:** Devanshi Patel, Jesse Mez, Badri N. Vardarajan, Lyndsay Staley, Jaeyoon Chung, Xiaoling Zhang, John J. Farrell, Michael J. Rynkiewicz, Lisa A. Cannon-Albright, Craig C. Teerlink, Jeffery Stevens, Christopher Corcoran, Josue D. Gonzalez Murcia, Oscar L. Lopez, Richard Mayeux, Jonathan L. Haines, Margaret A. Pericak-Vance, Gerard Schellenberg, John S. K. Kauwe, Kathryn L. Lunetta, Lindsay A. Farrer

**Affiliations:** 1 Department of Medicine (Biomedical Genetics), Boston University School of Medicine, Boston, Massachusetts; 2 Bioinformatics Graduate Program, Boston University, Boston, Massachusetts; 3 Department of Neurology, Boston University School of Medicine, Boston, Massachusetts; 4 Department of Neurology, Columbia University, New York, New York; 5 Department of Biology, Brigham Young University, Provo, Utah; 6 Department of Biostatistics, Boston University School of Public Health, Boston, Massachusetts; 7 Department of Physiology & Biophysics, Boston University School of Medicine, Boston, Massachusetts; 8 George E. Wahlen Department of Veterans Affairs Medical Center, Salt Lake City, Utah; 9 Huntsman Cancer Institute, Salt Lake City, Utah; 10 Department of Internal Medicine, University of Utah School of Medicine, Salt Lake City; 11 Department of Mathematics and Statistics, Utah State University, Logan; 12 Department of Neurology, University of Pittsburgh School of Medicine, Pittsburgh, Pennsylvania; 13 Department of Population & Quantitative Health Sciences, Case Western Reserve University School of Medicine, Cleveland, Ohio; 14 John P. Hussman Institute for Human Genomics, Miller School of Medicine, University of Miami, Miami, Florida; 15 Department of Pathology and Laboratory Medicine, University of Pennsylvania, Philadelphia; 16 Department of Ophthalmology, Boston University School of Medicine, Boston, Massachusetts; 17 Department of Epidemiology, Boston University School of Public Health, Boston, Massachusetts

## Abstract

**Importance:**

Some of the unexplained heritability of Alzheimer disease (AD) may be due to rare variants whose effects are not captured in genome-wide association studies because very large samples are needed to observe statistically significant associations.

**Objective:**

To identify genetic variants associated with AD risk using a nonstatistical approach.

**Design, Setting, and Participants:**

Genetic association study in which rare variants were identified by whole-exome sequencing in unrelated individuals of European ancestry from the Alzheimer’s Disease Sequencing Project (ADSP). Data were analyzed between March 2017 and September 2018.

**Main Outcomes and Measures:**

Minor alleles genome-wide and in 95 genes previously associated with AD, AD-related traits, or other dementias were tabulated and filtered for predicted functional impact and occurrence in participants with AD but not controls. Support for several findings was sought in a whole-exome sequencing data set comprising 19 affected relative pairs from Utah high-risk pedigrees and whole-genome sequencing data sets from the ADSP and Alzheimer’s Disease Neuroimaging Initiative.

**Results:**

Among 5617 participants with AD (3202 [57.0%] women; mean [SD] age, 76.4 [9.3] years) and 4594 controls (2719 [59.0%] women; mean [SD] age, 86.5 [4.5] years), a total of 24 variants with moderate or high functional impact from 19 genes were observed in 10 or more participants with AD but not in controls. These variants included a missense mutation (rs149307620 [p.A284T], n = 10) in *NOTCH3*, a gene in which coding mutations are associated with cerebral autosomal-dominant arteriopathy with subcortical infarcts and leukoencephalopathy (CADASIL), that was also identified in 1 participant with AD and 1 participant with mild cognitive impairment in the whole genome sequencing data sets. Four participants with AD carried the *TREM2* rs104894002 (p.Q33X) high-impact mutation that, in homozygous form, causes Nasu-Hakola disease, a rare disorder characterized by early-onset dementia and multifocal bone cysts, suggesting an intermediate inheritance model for the mutation. Compared with controls, participants with AD had a significantly higher burden of deleterious rare coding variants in dementia-associated genes (2314 vs 3354 cumulative variants, respectively; *P* = .006).

**Conclusions and Relevance:**

Different mutations in the same gene or variable dose of a mutation may be associated with result in distinct dementias. These findings suggest that minor differences in the structure or amount of protein may be associated with in different clinical outcomes. Understanding these genotype-phenotype associations may provide further insight into the pathogenic nature of the mutations, as well as offer clues for developing new therapeutic targets.

## Key Points

**Question:**

Can rare genetic variants for Alzheimer disease be identified using nonstatistical approaches?

**Findings:**

In this genetic association study, variants with high functional effect were observed in participants with Alzheimer disease but not in controls in *NOTCH3*, a gene previously associated with cerebral autosomal-dominant arteriopathy with subcortical infarcts and leukoencephalopathy (CADASIL), and *TREM2* (Q33X) that in homozygous form causes Nasu-Hakola disease.

**Meaning:**

Different mutations in the same gene or variable dose of a particular mutation may be associated with dissimilar types of dementia.

## Teaser

This genetic association study uses whole-exome sequencing to identify genetic variants associated with the risk of Alzheimer disease in Alzheimer’s Disease Sequencing Project participants of European ancestry.

## Introduction

Alzheimer disease (AD) is the most common type of dementia and affects an estimated 5.7 million individuals in the United States, with the number projected to rise to 14 million by 2050.^1^ Susceptibility to AD is highly heritable (*h*^2^ = 58%-79%),^2^ but only about one-third of the genetic component is accounted for by common variants discovered through genome-wide association studies.^2^ Some of the unexplained heritability of AD may be due to rare variants, which remain challenging to discover in genomic studies because of statistical power limitations, despite large sample sizes.^3^ Genome-wide searches have identified AD associations with rare variants in relatively few genes, including *TREM2*, *AKAP9*, *UNC5C*, *ZNF655*, *IGHG3*, and *CASP7*,^4,5,6,7,8^ and methods to evaluate rare variants are still under development.^3^ We applied a strategy focused on rare variants occurring only in cases to identify and characterize additional high-penetrance risk variants in AD that would be otherwise undetected in analyses that do not render results when a variant is not observed in the control group.

## Methods

### Study Population and Data Pipeline

The Alzheimer’s Disease Sequencing Project (ADSP) performed whole-exome sequencing (WES) on DNA samples obtained from participants of non-Hispanic European ancestry (EA) and a group of Caribbean Hispanic individuals that was deemed too small for inclusion in this study. A total of 5617 participants with AD met National Institute of Neurological and Communicative Diseases and Stroke/Alzheimer’s Disease and Related Disorders Association criteria for possible, probable, or definite AD after clinical and/or neuropathologic examination^9^ and 4594 controls were cognitively normal. The ADSP participants were selected using a risk score based on age, sex, and *APOE* ε4 carrier status to maximize cases most likely to have AD risk variants and controls most likely to have AD protective variants. Sample characteristics are provided in eTable 1 in the Supplement.

Written informed consent was obtained from all participants who were 60 years or older or from their authorized legal representative. This study was approved by the Boston University Institutional Review Board. Data were analyzed between March 2017 and September 2018. This study followed the Strengthening the Reporting of Genetic Association Studies (STREGA) reporting guideline.

### Gene Selection and Variant-Filtering Pipeline

Rare variants were analyzed under 2 different schemes: one considering all genes in the genome and another focused on genes for which there was prior evidence linking them to AD, AD-related endophenotypes, or other disorders in which adult-onset dementia was the cardinal feature. Selection of genes for the latter analysis scheme (listed in eTable 2 in the Supplement) was based on a review of the literature and required either genome-wide significant association findings or generally accepted functional evidence. Details about DNA sequencing, data quality control, and variant selection and annotation are provided in the eMethods and eTable 3 in the Supplement. The study design is illustrated in eFigure 1 in the Supplement.

### Rare Variant Analysis in an Independent Data Set

To extend and enhance the discovery of novel associations, we evaluated WES data obtained from 19 AD-affected first- or second-cousin pairs identified in the Utah Population Database belonging to a pedigree with a statistical excess of AD risk. These pedigrees are genealogically independent at least as far back as the early 1800s. Details of the Utah Population Database, case classification, and identification of high-risk pedigrees have been published elsewhere.^18^ A series of steps that included filters for sharing among affected relatives, frequency in several public next-generation sequencing databases, pathogenicity, and relevance to AD pathologic factors resulted in 130 variants exome wide for further evaluation (eMethods in the Supplement).

### Statistical Analysis

#### Haplotype Analysis

PLINK was used to find common single-nucleotide polymorphisms (SNPs) near the rare variant of interest within a specified kilobase (kb) window. The wildcard option was used to infer haplotypes and estimate haplotype frequencies.^10^ Haploview was used to visualize regional linkage disequilibrium and confirm haplotypes and frequencies among different SNP combinations using multimarker haplotype association tests.^11^

#### Protein Homologic Modeling and Pathway Analysis

Protein homologic modeling was performed for several high-impact variants in *NOTCH3* with BLAST-P, version 2.7.1,^12^ SWISS-MODEL, SMTL version 2019-02-13 (PDB release 2019-02-08),^13^ and Maestro, version 11.2 software.^14^ Additional details of the modeling procedures are provided in the eMethods in the Supplement. A high-confidence (confidence score >0.7) human protein-protein interaction network was then created with version 10 of the STRING database for *NOTCH3* and its ligand *JAG1*.^15^ The set of genes forming the protein network was tested for gene-set enrichment using Protein Analysis Through Evolutionary Relationships pathways and the Fisher exact test with false discovery rate multiple test correction.^16^

#### Estimation of Burden of Rare Variants

A gene-set test was performed to evaluate the burden of high- and moderate-impact mutations in the set of AD- or dementia-related genes among participants with AD compared with controls. Logistic regression models, including covariates for sex, age, sequencing center, and principal components of ancestry, were evaluated using the Combined and Multivariate Collapsing method^17^ in R, version 3.5.0 (R Foundation). Findings were considered significant at 2-tailed *P* < .05.

## Results

After performing data-filtering steps, 5617 participants with AD (3202 [57.0%] women; mean [SD] age, 76.4 [9.3] years) and 4594 controls (2719 [59.0%] women; mean [SD] age, 86.5 [4.5] years) remained for analysis.

### Rare Variants in *NOTCH3*

Evaluation of high- and moderate-impact rare variants in genes that were previously established as genetically or functionally associated with AD or dementia revealed a missense mutation in *NOTCH3* (rs149307620; p.A284T) that was present in 10 AD cases, but no controls (eTable 4 in the Supplement). This variant is rare in EAs (minor allele frequency [MAF], 0.0005)^19^ and was verified by Sanger sequencing in 8 of these participants for whom DNA was available. Because several other high- or moderate-impact *NOTCH3* mutations have been associated with cerebral autosomal-dominant arteriopathy with subcortical infarcts and leukoencephalopathy (CADASIL), a diagnostically distinct disorder marked by severe headaches in young adulthood followed by strokes and dementia later in life,^20^ we sought clinical and autopsy data from the participants with AD with the rs149307620 mutation to determine whether they are enriched for cerebrovascular risk factors. Neuropathologic information that was available for one of these participants revealed moderate atherosclerosis but no arteriosclerosis, lacunes, or microinfarcts, which are hallmarks of CADASIL (eTable 5 in the Supplement).

Autopsy also confirmed the presence of AD abnormalities (Consortium to Establish a Registry for Alzheimer’s Disease [CERAD] neuritic plaques, moderate; CERAD diffuse plaques, moderate; and Braak neurofibrillary degeneration, stage VI). The mean (SD) age at symptom onset for the 10 *NOTCH3* mutation carriers was 80.5 (6.7) years, which was similar to that for the entire sample of ADSP EA cases (80.9 [9.1] years) and greater than age at onset of cognitive impairment among individuals with CADASIL (usually <50 years). None of the *NOTCH3* mutation carriers had a history of clinical strokes (although 1 carrier had multiple infarcts shown on magnetic resonance imaging and a history of diabetes and cardiovascular disease) and all had prominent memory impairment as the initial presentation with a progressive course. One other *NOTCH3* mutation (rs114447350; p.P2074Q) was observed in 4 participants with AD but not in controls (eTable 4 in the Supplement). Unlike rs149307620, this variant is not rare in EAs (MAF, 0.024) or in persons of African ancestry (MAF, 0.091),^21^ suggesting it is unlikely to be pathogenic.

Because rs149307620 is rare, we investigated the possibility that this mutation occurred once or only a few times by performing a haplotype analysis with common SNPs. This analysis revealed a 5-SNP haplotype with a frequency of 15% in the participants with AD and 14% in controls that is common to all 10 cases with the *NOTCH3* mutation (eFigure 2 in the Supplement). The mean pairwise identical-by-descent (IBD) sharing for the 10 mutation carriers (mean [SD] π̂ = 0.028 [0.025]) is slightly larger than the mean pairwise IBD sharing within the rest of ADSP sample (mean [SD] π̂ = 0.013 [0.026]), indicating that the carriers are not more closely related to each other than to all participants. Taken together, these results suggest that the mutation in these participants originated in a common ancestor who lived many generations ago.

To investigate the possibility that the mutation carriers belong to a particular subpopulation, we plotted the first 2 principal components of ancestry that were derived previously for the entire sample^7^ and observed that 8 of the 10 mutation carriers were clustered in a distinct minor portion of the sample (eFigure 3 in the Supplement). Analysis of mitochondrial DNA variants revealed that most individuals in this cluster had mitochondrial haplogroups K1a1b1a or K1a9 that are common among Ashkenazi Jewish individuals.^22^ Moreover, *NOTCH3* mutations carriers accounted for 4.0% of the participants with AD who have either the K1a1b1a or K1a9 haplogroup. The proportion of mutation carriers in this cluster was significantly greater among participants with AD (8 of 358 [2.2%]) than controls (0 of 337) (*Z* = 2.76, *P* = .006). The frequency of the rs149307620 mutation is about 25 times higher in Ashkenazi Jewish individuals (MAF, 0.0046) compared with other EA groups (MAF, 0.00019).^19^

Analysis of the 130 variants that met the filtering criteria in the 19 affected cousin pairs from the Utah high-risk pedigrees provided additional support for a role of *NOTCH3* in AD. Both affected individuals in 1 family who are half-first cousins had 2 rare *NOTCH3* missense mutations—rs141402160 (p.G248A) and rs140914494 (p.A198E)—each with a population frequency of 0.0002.^20^ Review of available clinical and family history information for this family did not indicate findings consistent with CADASIL in the probands or relatives. The pedigree of the carriers of the rs141402160 and rs140914494 mutations includes 7 additional members who had AD or dementia listed on death certificates (eFigure 4 in the Supplement). All but 1 of the individuals in the line of descent from the common ancestor of the pair died before age 60 years, prior to the age at onset of AD in the cases. An affected cousin pair in an independent family had the *NOTCH3* missense variant rs112197217 (p.H1133Q), which has a frequency of 0.010 in EAs,^21^ but is rarer in other populations (eFigure 5 in the Supplement). The evidence of AD among other relatives in this family was inconclusive.

To further distinguish which of the 5 *NOTCH3* variants identified in the ADSP WES sample and Utah families may be related to AD, we screened for these variants in whole-genome sequence (WGS) data obtained from a multiethnic sample of unrelated 1432 participants with AD and 1660 controls in the ADSP Extension Study, 550 participants with AD and 283 controls in the ADSP multiethnic Family Study,^23^ and 809 participants in the Alzheimer’s Disease Neuroimaging Initiative Study (239 participants with AD, 321 participants with mild cognitive impairment, 249 controls).^24^ Characteristics of participants in these data sets are provided in eTable 6 in the Supplement. The minor alleles for rs11219217 (n = 70) and rs114447350 (n = 286) were observed appreciably in participants with AD, participants with mild cognitive impairment, and controls of multiple ethnicities suggesting that they are not associated with AD risk. The rs149307620 variant was found in 1 AD case (age at onset, 89 years) and 1 mild cognitive impairment case (age at onset, 76 years), but not in controls. The rare rs141402160 and rs140914494 variants were not detected in any of the WGS samples.

**Figure 1.**
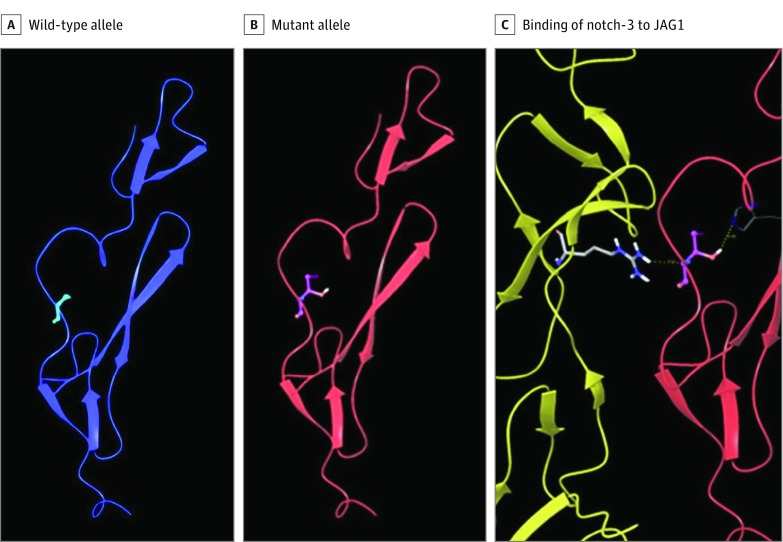
Notch-3 Protein Model Highlighting Position of the Alzheimer Disease–Associated Single-Nucleotide Polymorphism rs149307620 (p.A284T) Predicted model for wild-type allele with alanine at mutation site (A), mutant allele with threonine at mutation site (B), and binding of notch-3 (red) to JAG1 ligand (yellow) (C). Possible hydrogen bonding that would likely cause greater interaction with the ligand is displayed.

Protein modeling showed that the rs149307620 mutation is located in the EGF repeat region between EGF10 and EGF11 and more precisely in the EGF calcium binding (EGF_CA) domain, near the Jagged-1 (*JAG1*)-*NOTCH3* binding site.^25^ Modeling predicted that the major allele for rs149307620 results in wild-type notch-3 with a corresponding amino acid alanine (Figure 1A). The alanine side chain is nonpolar and would not be predicted to have any intraprotein or interprotein interactions. The minor allele for rs149307620 results in mutant notch-3 with a corresponding amino acid threonine (Figure 1B). Threonine is polar and will form hydrogen bonds where possible with itself or with a polar histidine nearby in *NOTCH3*. This action then alters the backbone conformation in this region in the model and allows additional interactions with a polar arginine at the site of *JAG1-NOTCH3* binding (Figure 1C). These results suggest that the mutant Notch-3 causes greater interaction with the ligand, possibly changing downstream processes. The other AD-associated *NOTCH3* mutations, rs141402160 and rs140914494, also involve either the gain or loss of an alanine. In both instances, the mutation change leads to increased polarity and hydrogen bonding with possible increased interactions to a greater or lesser extent that observed with rs149307620.

**Figure 2.**
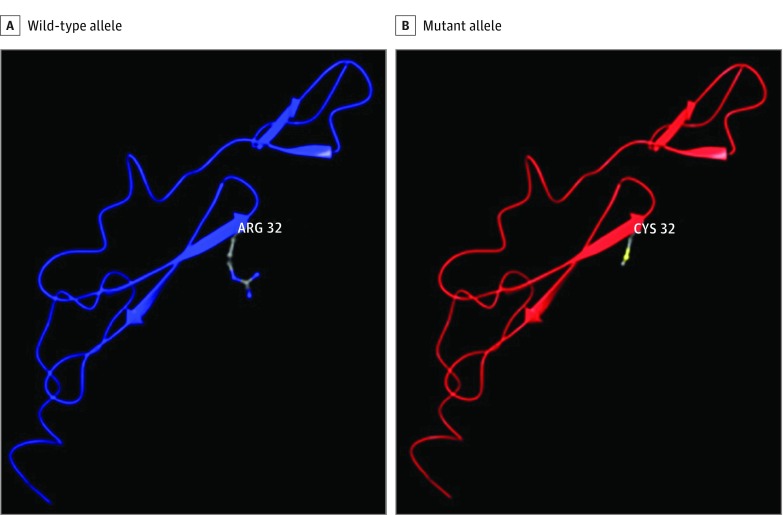
Homologous Protein Modeling of Cerebral Autosomal-Dominant Arteriopathy With Subcortical Infarcts and Leukoencephalopathy (CADASIL) *NOTCH3* rs137852641 (p.R332C) Mutation Predicted model for wild-type allele with arginine at the mutation site (A) and mutant allele with cysteine at the mutation site (B). Gain of a cysteine residue disrupts disulfide bonding within the protein, affecting overall protein structure.

Unlike the rs149307620 and rs141402160 mutations, but similar to the rs140914494 mutation, most of the more than 25 reported distinct *NOTCH3* mutations causing CADASIL are located in exons 3 and 4 (Table 1).^26^ However, 1 CADASIL-associated variant, rs137852641, is a missense mutation in codon 332 in exon 6, resulting in the replacement of an arginine residue with a cysteine^27^ that is proximate to rs149307620 (codon 284 in exon 6) (Figure 2).

**Table 1.** *NOTCH3* Mutations With Predicted Functional Impact on AD Risk

SNP	Position (chr 19)^a^	Exon	GnomAD Frequency	Protein Position	Residue Change	Observed Mutation Carriers
rs140914494	15 192 046	4	0.00003	198	Ala>Glu	AD-affected relative pair
rs141402160	15 191 804	5	0.00005	248	Gly>Ala	AD-affected relative pair
rs149307620	15 191 610	6	0.00029	284	Ala>Thr	11 Participants with AD, 1 participant with MCI

Abbreviations: AD, Alzheimer disease; GnomAD, Genome Aggregation Database; MCI, mild cognitive impairment; SNP, single-nucleotide polymorphism.

^a^ Chromosome position according to GRCh38.p12 assembly.

To further explore the biological functions and pathways for *NOTCH3* in AD, a high-confidence protein-protein interaction network was constructed including *NOTCH3* and *JAG1*. The resulting 30-gene interaction network contains several AD-related genes, including *BACE1*, *PSEN1*, *PSEN2*, and *APP* (eFigure 6 in the Supplement). Gene-set enrichment analysis revealed that the network genes were significantly enriched in the Notch signaling pathway (*P* = 6.48 × 10^−49^), angiogenesis (*P* = 1.61 × 10^−12^), and 2 AD-related pathways involving secretase-mediated amyloid precursor protein cleavage (*P* = 3.50 × 10^−16^) and presenilin γ-secretase complex (*P* = 5.78 × 10^−26^) (Table 2).

**Table 2.** Gene-Set Enrichment Analysis of NOTCH3/JAG1 Protein-Protein Interaction Network

PANTHER Pathway	No. of Genes Annotated to Pathway	No. of Genes in Network	Expected *P* Value^a^	Fold Enrichment	Unadjusted *P* Value	FDR
Notch signaling	42	22	.06	>100	3.98 × 10^−51^	6.48 × 10^−49^
Presenilin	123	14	.18	>100	7.09 × 10^−28^	5.78 × 10^−26^
Amyloid secretase	69	7	.10	>100	8.59 × 10^−18^	3.50 × 10^−16^
Angiogenesis	173	10	.25	40.54	4.92 × 10^−14^	1.61 × 10^−12^

Abbreviations: FDR, false discovery rate; PANTHER, Protein Analysis Through Evolutionary Relationships classification system.

^a^ Expected probability of observing at least x number of genes out of the total number of genes in the PANTHER list annotated to a particular pathway, given the proportion of genes in the reference *Homo sapiens* whole genome that are annotated to that pathway.

### *TREM2* Q33X

We also identified the high-impact *TREM2* rs104894002 (p.Q33X) mutation in 4 of 5617 (0.071%) participants with AD (eTable 4 in the Supplement), a frequency that is slightly lower than that observed in a *TREM2* sequencing study of participants with AD and controls in 2013 (2 of 1084 [0.17%] participants with AD).^4^ Because this mutation in a homozygous state causes Nasu-Hakola disease, a rare autosomal-recessive disorder characterized by early-onset dementia and multifocal bone cysts,^28^ we evaluated clinical data obtained from the 4 *TREM2* Q33X mutation carriers to assess potential pleiotropic effects (eTable 7 in the Supplement). All of these participants met the criteria for probable AD and none had reported bone cysts or unusual behavioral symptoms.

### Other Rare Mutations

A total of 32 moderate- or high-impact variants in 24 previously established genes for AD or other dementias were each observed in 4 or more participants with AD and no controls (eTable 4 in the Supplement). Five of these variants were previously reported as associated with AD and include missense mutations in *PSEN1* (rs63749824/p.A75V [n = 7]^29^; rs63750592/p.R35Q [n = 4]),^30^ *SORL1* (rs139710266/p.Y391C [n = 5]),^31^ and *MAPT* (rs63750424/p.R741W [n = 4]),^32^ and a stop-gain mutation in *ABCA7* (rs145987355/ p.E1679X [n = 4]).^33^ Genome-wide, 24 variants in 19 genes with moderate to high functional impact were observed in 10 or more participants with AD but absent in controls (eTable 8 in the Supplement). Further examination of the genes represented by multiple variants revealed that 10 participants had 3 *ABCD4* missense variants (rs57773157/p.G248, rs34992370/p.V172I, rs58272575/p.G59R) that co-occur in a rare 8-SNP haplotype spanning 12.9 kb with a frequency of 0.3% in cases and 0% in controls (eFigure 7A in the Supplement). Another 10 participants had 2 *CELSR1* missense variants (rs61741871/p.2983A and rs75983687/p.2703M), and 8 of these participants also had 2 *GTSE1* missense variants (rs34404175/p.A219V and rs35503220/p.A293A). One participant was homozygous for all 4 variants. The participants who had these *CELSR1* and *GTSE1* variants share a rare 12-SNP haplotype spanning 77.6 kb with a frequency of 0.1% in cases and 0.1% in controls (eFigure 7B in the Supplement). Estimates of IBD sharing for the 10 participants with the *ABCD4* variants were only slightly higher (mean [SD] π̂ = 0.015 [0.028]) and for the 8 participants with AD and the *CELSR1* and *GTSE1* variants (mean [SD] π̂ = 0.008 [0.015]) were lower than genome-wide IBD sharing, suggesting that they are not more closely related to each other than to all participants. There were few common SNPs in the 500-kb region including the rare *ABCD4* variants, suggesting high-sequence conservation in this region.

To identify additional genes that may have overrepresentation of deleterious AD-related variants, we filtered genes that contained at least 3 distinct variants, each occurring in at least 5 participants with AD but absent in controls (eTable 9 in the Supplement). The *ABCD4* rs61744947/p.P2983A variant appears on the same haplotype containing the other 3 *ABCD4* variants. Three *LAMC3* variants were observed in the same 7 participants. *TTN* had the greatest number of distinct variants (n = 6) that were observed in participants with AD only. Genome-wide, 9 genes not previously associated with AD contained a high functional impact variant that was present in at least 7 participants with AD but absent in controls (eTable 10 in the Supplement).

### Rare Variant Burden

To test if AD is associated with greater burden of rare deleterious variants, gene burden tests were performed for models including high-impact variants and high- and moderate-impact variants for MAF of 0.01 or lower and MAF of 0.5 or lower in genes previously associated with AD risk, AD-related traits, or other dementias (Table 3). These analyses showed that participants with AD had a significantly higher burden of moderate- and high-impact rare deleterious variants in this group of genes compared with controls (2314 vs 3354 cumulative variants, respectively; *P* = .006).

**Table 3.** Rare Variant Burden for Established Alzheimer Disease Genes

Model	β (SE)	*P* Value
High impact, MAF ≤ 0.01	0.005 (0.166)	.98
High/moderate impact, MAF ≤ 0.01	0.062 (0.023)	.006
High impact, MAF ≤ 0.05^a^	0.005 (0.166)	.98
High/moderate impact, MAF ≤ 0.05^a^	0.061 (0.022)	.006

Abbreviation: MAF, minor allele frequency.

^a^ No deleterious variants with MAF between 0.01 and 0.05 were observed in this group of genes.

## Discussion

We identified several rare variants that have a high probability of damage to protein structure and may increase AD risk. These variants were not detected in previous analyses of the same ADSP WES data set that were agnostic with respect to functional impact of the variants and conducted using current statistical testing approaches.^7^ Our focus on variants observed in participants with AD but not controls yielded results that are often undetected by traditional genetic association methods that cannot evaluate empty cells, regardless of sample size or frequency of variants among cases. Several of our top-ranked results confirm previously identified AD associations with rare variants, including *PSEN1* rs63749824^29^ and rs63750592,^30^ *SORL1* rs139710266,^31^ *MAPT* rs63750424,^32^ and *ABCA7* rs145987355,^33^ which suggest that novel findings identified by our approach may be robust. Two of our novel findings offer additional evidence of shared genetic mechanisms between AD and other rare dementia syndromes, namely CADASIL and Nasu-Hakola disease. Our study also suggests that participants with AD have a significantly higher burden of deleterious rare coding variants in known AD, AD-related, or other dementia genes compared with controls. This observation generalizes previous findings in *SORL1*,^31,34,35^ *MAPT*,^32,36^ *TREM2*,^4,37,38^ and *ABCA7*^33,39,40,41^ that both common and rare variants in the same gene may independently contribute to AD risk.

We observed the rare *NOTCH3* rs149307620 allele in 11 participants with AD and 1 participant with mild cognitive impairment, but not in controls, in the combined ADSP WES, ADSP WGS, and Alzheimer’s Disease Neuroimaging Initiative WGS data sets. The most remarkable finding from analysis of the Utah high-risk pedigree WES data set was rare *NOTCH3* rs140914494 and rs141402160 alleles in a pair of affected half first-cousins. These mutations in exons 4 (rs140914494), 5 (rs141402160), and 6 (rs149307620) are located in the JAG1 binding site and involve the gain or loss of an alanine residue (Table 1). Based on this evidence alone, it is unclear whether one or both of the rs140914494 and rs141402160 mutations, which are likely in *cis* given their probable inheritance from a single common ancestor, have a role in AD. In contrast, the rs114447350 and rs112197217 variants are located near the end of the coding sequence (exons 33 and 21, respectively) and may be clinically benign^42^ and thus unlikely to be causally related to AD. Many other *NOTCH3* variants have been associated with CADASIL that typically replace the wild-type amino acid with a cysteine residue or replace a highly conserved cysteine residue with another amino acid,^20,26,27^ although there are several exceptions.^43^ Available clinical and autopsy data for the individuals with *NOTCH3* mutations were consistent with the diagnosis of AD and not CADASIL. Our protein modeling demonstrated that the AD-associated *NOTCH3* mutations in exons 4 to 6 result in quantitative changes in hydrogen bonding causing increased ligand interaction, whereas CADASIL *NOTCH3* mutations lead to qualitative changes involving disrupted disulfide bonding that affect overall protein structure and receptor maturation and differ with respect to their consequences on both ligand binding and ligand-induced signaling.^26,27^

Our protein-protein interaction network and gene-set enrichment analyses demonstrated that *NOTCH3* is associated with AD pathways and biological processes. Notch-3 signaling can be triggered by both delta-JAG-type ligands and requires ADAM10 and presenilin-1 or -2, making it part of the AD-related presenilin pathway.^44^ Previous studies showed that JAG1-Notch signaling and subsequent hippocampal neurogenesis and astrogenesis are regulated by cleavage by *BACE1*, a promising AD drug target.^44^ This process, which is more active during early development and decreases in adulthood, affects normal neuronal development and alters neurogenesis and thus can have long-term effects.^44^ In addition, Notch-3 is a substrate for γ-secretase (presenilin) inhibition, which, when dysregulated, can cause misprocessing of the amyloid precursor protein resulting in accumulation of the toxic amyloid-β peptide.^45^

To our knowledge, these collective genetic and bioinformatics findings provide the strongest possible pathogenic link to date between *NOTCH3* and AD. A previous study reported an association of AD with a distinct *NOTCH3* mutation (p.R1231C) in a Turkish family^46^; however, this variant was detected in only 1 affected member and there is conflicting information about its pathogenicity.^47^ Sassi et al^48^ tested the hypothesis that genes associated with mendelian adult-onset leukodystrophy are also associated with AD in a sample including 332 sporadic participants with AD and 676 controls and found a significant gene-based association with *NOTCH3*, a result driven primarily by a common synonymous coding variant.

The *TREM2* Q33X mutation that was observed in 4 participants with AD in our sample and in 4 participants with AD and 1 unaffected relative with an unspecified age in gene-resequencing studies targeting *TREM2*^4,49,50^ is rarer than the well-documented R47H variant that has been associated with increased risk of AD in several studies,^4,51^ including the ADSP cohort.^7,8^ Homozygosity of this mutation causes Nasu-Hakola disease, a rare disorder characterized by early-onset dementia and multifocal bone cysts,^28^ and has also been observed in a member of a Turkish family with frontotemporal dementia–like syndrome, including the appearance of aggressive behavior and generalized tonic-clonic seizures before age 30 years but without bone involvement.^52^ Because persons with Nasu-Hakola disease and the frontotemporal dementia syndrome case have a more severe phenotype overall and much earlier onset of dementia symptoms than participants with late-onset AD who are heterozygous for Q33X, the behavior of this mutation may more resemble an intermediate inheritance than an autosomal-dominant model. This idea is consistent with the observation that both living parents of a patient with Nasu-Hakola disease who were obligate Q33X heterozygotes had evidence of β-amyloid deposition by cerebrospinal fluid analysis and florbetapir positron emission tomographic imaging.^52^

Furthermore, unlike the *TREM2* R47H mutation and rare coding variants at other loci that have been associated with AD,^4,5,6,7,8^ the *NOTCH3* rs149307620 and *TREM2* Q33X mutations appear to be fully penetrant among persons surviving to late age, which perhaps would be the first examples of causative mutations for late-onset AD. This assertion is somewhat speculative given the small number of participants with AD documented to have these mutations.

Our study also implicated multiple functional variants in several novel genes as risk factors for AD. Mutations in *ABCD4* cause an inborn error of vitamin B_12_ metabolism.^53^ Vitamin B_12_ deficiency is associated with cognitive impairment, and the level of circulating vitamin B_12_ has been associated with AD risk.^54^ *ABCD4* encodes an adenosine triphosphate–binding cassette transporter that is in the same family as well-established AD gene *ABCA7*.^39,40^ The AD-associated *CELSR1* rs61741871 (P2983A) missense variant has also been associated with craniorachischisis, which is a severe neural tube defect,^55^ and other *CELSR1* variants have been identified as ischemic stroke risk factors in Japanese individuals.^56^ The *CELSR1-3* family of genes has multiple functions in the nervous system and distinct roles in brain development and maintenance.^57^ *GTSE1* regulates G1/S cell cycle transition and microtubule stability and is involved in pivotal neurodegeneration pathways.^58^ It is not clear which of these *CELSR1* and *GTSE1* mutations may directly influence AD risk. *LAMC3* encodes laminin subunit γ 3, and multiple experimental studies have linked laminins to AD.^59,60^ *LAMC3* has been significantly associated with age at onset of AD.^61^

### Limitations

Our study has several limitations. Because we focused on rare variants, our sample of more than 10 000 participants was inadequate to establish statistical significance. Thus, our findings require replication in independent samples. We were unable to replicate these findings in the Alzheimer’s Disease Genetics Consortium genome-wide association study data set because of low and inconsistent imputation quality for these rare variants, despite the use of the large Haplotype Reference Consortium reference panel.^62^ In addition, our genome-wide MAC cutoffs for focusing on particular variants were arbitrary and, therefore, some important findings may have been overlooked.

It is possible that cryptic relatedness in the sample may have exaggerated some of our results; however, among the highlighted findings, the largest pairwise IBD score of 0.11 (indicating an association slightly more distant than first-cousins) was observed for 1 pair of *NOTCH3* mutation carriers. Our scheme for selecting genes previously associated with AD, AD-related traits, or other dementias omitted important loci that were reported after we completed most of our analyses (eg, *ADAM17*^63^), ascertained through a connection to a nondementing illness (eg, *TBK1*^64^), or do not have variants linked to late-onset AD (eg, *TYROBP*^65^). In addition, because we were unable to validate all rare variants reported in this study owing, in part, to availability of specimens containing these variants, some of the highlighted associations may be false-positives due to variant calling errors. However, most of these variants, including *TREM2* Q33X and all of those in *ABCD4*, *CELSR1*, and *GTSE1*, have been reported previously.^21^

Although one of the explicit goals of the ADSP is to identify variants that protect against AD,^23^ the design corresponding to the one we used to identify risk variants (ie, a controls-only analysis) is less rigorous because, in the absence of statistically significant tests, it is difficult to demonstrate a protective effect if the variant has reduced penetrance.

### Conclusions 

We observed associations with novel variants in previously established AD genes and with several novel potential AD genes that did not emerge in previous analyses of a large WES data set using conventional statistical thresholds.^7^ Several of the results implicating novel AD genes—in particular, *ABCD4, CELSR1, GTSE1*—merit further epidemiologic and experimental studies. Our findings with the *NOTCH3* and *TREM2* variants suggest that mutations in the same gene can result in dissimilar types of dementia. Moreover, a variable dose of a particular mutation (ie, *TREM2* Q33X) can cause different types of dementia. These findings suggest that minor differences in protein structure or amount of wild-type protein can result in different clinical outcomes. Understanding these genotype-phenotype associations may provide further insight into the pathogenic nature of the mutations, as well as offer clues for developing new therapeutic targets.

## Notes

Supplement. **eMethods.** Detailed Methodology **eReferences** **eTable 1.** Characteristics of Subjects in the ADSP WES Case-Control Dataset **eTable 2.** Previously Established Genes for AD, AD-Related Traits and Other Dementias **eTable 3.** Filtering Pipeline of Rare Variants **eTable 4.** High and Moderate Impact Rare Variants in Previously Established AD Genes Occurring In ≥ 4 Participants With AD and No Controls **eTable 5.** Characteristics of AD Subjects in the ADSP WES Dataset With the *NOTCH3* rs149307620 Mutation **eTable 6.** Characteristics of Subjects in the WGS Replication Datasets **eTable 7.** Characteristics of AD Subjects With the *TREM2* rs104894002 Mutation (Q33X) **eTable 8.** High and Moderate Impact Rare Variants Genome-Wide Occurring in > 10 Participants With AD and No Controls **eTable 9.** Genes With ≥ 3 Distinct High/Moderate Disease Impact Rare Variants Each With a MAC ≥ 5 and Occurring Only in Cases **eTable 10.** High Impact Rare Variants Genome-Wide With a MAC ≥ 7 and Occurring Only in AD Cases **eFigure 1.** Study Design **eFigure 2.** Haplotype Analysis of the Rare *NOTCH3* rs149307620 Variant **eFigure 3.** Population Substructure of the ADSP Discovery Sample **eFigure 4.** Utah Pedigree Segregating rs141402160 and rs140914494 Mutations **eFigure 5.** Utah Pedigree Segregating rs112197217 Mutation **eFigure 6.** Protein-Protein Interaction Network Including *NOTCH3* and *JAG1* **eFigure 7.** Haplotype Analysis of *ABCD4* and *CELSR1/GTSE1*
