# Errors in Results and Supplement

**DOI:** 10.1001/jamanetworkopen.2020.23811

**Published:** 2020-09-23

In the Original Investigation titled “Association of Rare Coding Mutations With Alzheimer Disease and Other Dementias Among Adults of European Ancestry,”^1^ published March 29, 2019, there were errors in the Results section and errors in the Supplement. In the Results, the variant given as p.A293A should have been p.A293T, and the variant given as p.P2938 should have been p.A128V. This article has been corrected.^1^
