# Supplementary material for: Association of Rare Coding Mutations With Alzheimer Disease and Other Dementias Among Adults of European Ancestry
Source: JAMA Netw Open. 2019 Mar 29;2(3):e191350. doi: 10.1001/jamanetworkopen.2019.1350 (PMC6450321; doi:10.1001/jamanetworkopen.2019.1350)
Supplement: Supplement. — eMethods. Detailed Methodology eReferences eTable 1. Characteristics of Subjects in the ADSP WES Case-Control Dataset eTable 2. Previously Established Genes for AD, AD-Related Traits and Other Dementias eTable 3. Filtering Pipeline of Rare Variants eTable 4. High and Moderate Impact Rare Variants in Previously Established AD Genes Occurring In ≥ 4 Participants With AD and No Controls eTable 5. Characteristics of AD Subjects in the ADSP WES Dataset With the NOTCH3 rs149307620 Mutation eTable 6. Characteristics of Subjects in the WGS Replication Datasets eTable 7. Characteristics of AD Subjects With the TREM2 rs104894002 Mutation (Q33X) eTable 8. High and Moderate Impact Rare Variants Genome-Wide Occurring in > 10 Participants With AD and No Controls eTable 9. Genes With ≥ 3 Distinct High/Moderate Disease Impact Rare Variants Each With a MAC ≥ 5 and Occurring Only in Cases eTable 10. High Impact Rare Variants Genome-Wide With a MAC ≥ 7 and Occurring Only in AD Cases eFigure 1. Study Design eFigure 2. Haplotype Analysis of the Rare NOTCH3 rs149307620 Variant eFigure 3. Population Substructure of the ADSP Discovery Sample eFigure 4. Utah Pedigree Segregating rs141402160 and rs140914494 Mutations eFigure 5. Utah Pedigree Segregating rs112197217 Mutation eFigure 6. Protein-Protein Interaction Network Including NOTCH3 and JAG1 eFigure 7. Haplotype Analysis of ABCD4 and CELSR1/GTSE1 [file jamanetwopen-2-e191350-s001.pdf]

Supplementary Online Content

Patel D, Mez J, Vardarajan BN, et al; Alzheimer Disease Sequencing Project. Association of rare coding mutations with Alzheimer disease and other dementias among adults of European ancestry. *JAMA Netw Open*. 2019;2(3):e191350. doi:10.1001/jamanetworkopen.2019.1350

eMethods. Detailed Methodology

eReferences

eTable 1. Characteristics of Subjects in the ADSP WES Case-Control Dataset

eTable 2. Previously Established Genes for AD, AD-Related Traits and Other Dementias

eTable 3. Filtering Pipeline of Rare Variants

eTable 4. High and Moderate Impact Rare Variants in Previously Established AD Genes Occurring In ≥ 4 Participants With AD and No Controls

eTable 5. Characteristics of AD Subjects in the ADSP WES Dataset With the *NOTCH3* rs149307620 Mutation

eTable 6. Characteristics of Subjects in the WGS Replication Datasets

eTable 7. Characteristics of AD Subjects With the *TREM2* rs104894002 Mutation (Q33X)

eTable 8. High and Moderate Impact Rare Variants Genome-Wide Occurring in > 10 Participants With AD and No Controls

eTable 9. Genes With ≥ 3 Distinct High/Moderate Disease Impact Rare Variants Each With a MAC ≥ 5 and Occurring Only in Cases

eTable 10. High Impact Rare Variants Genome-Wide With a MAC ≥ 7 and Occurring Only in AD Cases

eFigure 1. Study Design

eFigure 2. Haplotype Analysis of the Rare *NOTCH3* rs149307620 Variant

eFigure 3. Population Substructure of the ADSP Discovery Sample

eFigure 4. Utah Pedigree Segregating rs141402160 and rs140914494 Mutations

eFigure 5. Utah Pedigree Segregating rs112197217 Mutation

eFigure 6. Protein-Protein Interaction Network Including *NOTCH3* and *JAG1*

eFigure 7. Haplotype Analysis of *ABCD4* and *CELSR1/GTSE1*

This supplementary material has been provided by the authors to give readers additional information about their work.

© 2019 Patel D et al. *JAMA Network Open.*

eMethods. Detailed Methodology

Data Processing

DNA sequencing was performed at the Broad Institute, the Baylor College of Medicine's Human Genome Sequencing Center, and Washington University's McDonnell Genome Institute. Genotypes for bi-allelic single nucleotide variants (SNVs) and short insertion-deletion polymorphisms (indels) were called using ATLAS2.[1] Only variants that overlapped the target regions captured by kits used by the three sequencing centers (Illumina and Nimblegen) were included. Variants that showed extreme departure from Hardy-Weinberg equilibrium (HWE, $P<10^{-6}$) among unrelated controls, were monomorphic, or had call rates <80% or average read depth <10 were excluded. In addition, samples that were outliers according to population substructure analysis or had a genetically determined sex that was inconsistent with the reported sex were excluded.[2] Cryptic relatedness was estimated using pairwise identity-by-descent (IBD) in PLINK[3] and one member from each of 69 pairs of individuals with $\hat{\pi}$ (proportion of alleles shared IBD)>0.4 was excluded.[2] After quality control (QC), there remained for analysis a total of 10,211 EA (5,617 AD cases, 4,594 controls) and 400 CH (221 cases, 179 controls) subjects.

Variant Selection and Annotation

Minor allele counts (MAC) genome-wide and in a group of 95 genes previously associated with AD, AD-related traits, or dementia by genetic association or experimental approaches (eTable 2 in the Supplement) were tabulated for rare variants occurring only in AD cases. Variants were annotated with the Variant Effect Predictor tool (VEP)[4] and Combined Annotation Dependent Depletion (CADD) scores[5] as having high functional impact (includes splice acceptor, splice donor, stop gained, frameshift, stop lost, start lost, or transcript amplification variants) or moderate functional impact (includes in-frame insertion, in-frame deletion, missense, or protein altering variants). A scaled CADD score of 20, for example, means that a variant is among the top 1% of deleterious variants in the human genome, and a scaled CADD score of 30 means that the variant is in the top 0.1%. Synonymous mutations and variants with a MAC < 3 were excluded. The filtering of variants at each step in the pipeline is shown in eTable 3 in the Supplement.

Variant Filtering Pipeline in Utah Pedigrees

We retained 564 rare variants that were shared between at least one cousin pair. We then retained all markers with a minor allele frequency (MAF) less than 0.1% in one of the following public whole exome and whole genome sequencing databases: 1000 Genomes,[6] Exome Aggregation Consortium (ExAC),[7] the Genome Aggregation Database (gnomAD),[7] and NHLBI GO Exome Sequencing Project (ESP),[8] resulting in the retention of 400 candidate variants. Variants that did not meet criteria for pathogenicity based on (1) American College of Medical Genetics and Genomics criteria[9] for "pathogenic" or "likely pathogenic", (2) association with loss or gain of gene function, and (3) prediction of haploinsufficiency were excluded. The genes containing the remaining 389 variants were screened for relevance to AD or β-amyloid pathology, after which 118 variants remained. Twelve variants were added to the group of 118 variants for further evaluation that were (1) reported in the literature as an AD risk variant (two variants), (2) had a p value < 0.09 for association of increased risk of AD in the ADGC GWAS (two variants),[10] (3) absent in whole genome sequences of 1,354 participants of the Wellderly cohort who are >80 years old with no chronic diseases and who are not taking chronic medications,[11] suggesting that the variant is not observed in the healthy elderly population (three variants), or (4) observed in multiple cousin pairs (five variants).

Protein Homology Modeling

Since the *NOTCH3* structure could not be found in any protein databases, homologous proteins were considered for modeling the region containing the *NOTCH3* mutations. Considering the AD-associated mutation on exon 6 (rs149307620), the closest matching sequence was identified in *NOTCH1* that had 76% homology of amino acid sequence identity suggesting that these proteins may have similar functions and it is a high

© 2019 Patel D et al. *JAMA Network Open.*

quality model. SWISS-MODEL was used to create a model for the *NOTCH3* region using homologous Protein Data Bank (PDB) structure 5UK5 in *NOTCH1*.[12] The same procedure was applied for other mutations in *NOTCH3* that are associated with Cerebral Autosomal Dominant Arteriopathy with Subcortical Infarcts and Leukoencephalopathy (CADASIL).

© 2019 Patel D et al. *JAMA Network Open.*

eReferences

1. Challis D, Yu J, Evani US, et al. An integrative variant analysis suite for whole exome next-generation sequencing data. *BMC Bioinformatics* 2012; 13:8.
2. Bis J, Jian X, Kunkle B, *et al*. Whole exome sequencing study identifies novel rare and common Alzheimer's-associated variants involved in immune response and transcriptional regulation. *Mol Psychiatry* 2018. doi:10.1038/s41380-018-0112-7.
3. Purcell S, Neale B, Todd-Brown K, *et al*. PLINK: a tool set for whole-genome association and population-based linkage analyses. *Am J Hum Genet* 2007; 81:559-575.
4. McLaren W, Gil L, Hunt SE, *et al*. The Ensembl Variant Effect Predictor. *Genome Biol* 2016; 17:122.
5. Kircher M, Witten DM, Jain P, O'Roak BJ, Cooper GM, Shendure J. A general framework for estimating the relative pathogenicity of human genetic variants. *Nat Genet* 2014; 46:310-315.
6. Auton A, Brooks LD, Durbin RM, *et al*. A global reference for human genetic variation. *Nature* 2015; 526:68-74.
7. Lek M, Karczewski KJ, Minikel EV, *et al*. Analysis of protein-coding genetic variation in 60,706 humans. *Nature* 2016; 536:285-291.
8. NHLBI GO Exome Sequencing Project (ESP) Exome Variant Server. http://evs.gs.washington.edu/EVS/
9. Richards S, Aziz N, Bale S, *et al*. Standards and guidelines for the interpretation of sequence variants: a joint consensus recommendation of the American College of Medical Genetics and Genomics and the Association for Molecular Pathology. *Genet Med* 2015; 17:405-424.
10. Naj AC, Jun G, Beecham GW, *et al*. Common variants at MS4A4/MS4A6E, CD2AP, CD33 and EPHA1 are associated with late-onset Alzheimer's disease. *Nat Genet* 2011; 43:436-441.
11. Erikson GA, Bodian DL, Rueda M, *et al*. Whole-genome sequencing of a healthy aging cohort. *Cell* 2016; 165:1002-1011.
12. Luca VC, Kim BC, Ge C, et al. Notch-Jagged complex structure implicates a catch bond in tuning ligand sensitivity. Science 2017; 355:1320-1324.
13. Reitz C, Jun G, Naj A, *et al*. Variants in the ATP-binding cassette transporter (ABCA7), apolipoprotein E ϵ4,and the risk of late-onset Alzheimer disease in African Americans. *JAMA* 2013; 309:1483-1492.
14. Hollingworth P, Harold D, Sims R, *et al*. Common variants at ABCA7, MS4A6A/MS4A4E, EPHA1, CD33 and CD2AP are associated with Alzheimer's disease. *Nat Genet* 2011; 43:429-435.
15. Beecham GW, Hamilton K, Naj AC, *et al*. Genome-wide association meta-analysis of neuropathologic features of Alzheimer's disease and related dementias. *PLoS Genet* 2014; 10:e1004606.
16. Sims R, van der Lee SJ, Naj AC, *et al*. Rare coding variants in PLCG2, ABI3, and TREM2 implicate microglial-mediated innate immunity in Alzheimer's disease. *Nat Genet* 2017; 49:1373-1384.
17. Meng Y, Baldwin CT, Bowirrat A, *et al*. Association of polymorphisms in the angiotensin-converting enzyme gene with Alzheimer disease in an Israeli Arab community. *Am J Hum Genet* 2006; 78:871-877.
18. Sogorb-Esteve A, García-Ayllón M, Gobom J, *et al*. Levels of ADAM10 are reduced in Alzheimer's disease CSF. *J Neuroinflammation* 2018; 15:213.
19. Yuan X, Sun S, Tan C, Yu J, Tan L. The role of ADAM10 in Alzheimer's disease. *J Alzheimers Dis* 2017; 58:303-322.
20. Gurses MS, Ural MN, Gulec MA, Akyol O, Akyol S. Pathophysiological function of ADAMTS enzymes on molecular mechanism of Alzheimer's disease. *Aging Dis* 2016; 7:479-490.
21. Chung J, Zhang X, Allen M, *et al*. Genome-wide pleiotropy analysis of neuropathological traits related to Alzheimer disease. *Alzheimers Res Ther* 2018; 10:22.
22. Logue MW, Schu M, Vardarajan BN, *et al*. Two rare AKAP9 variants are associated with Alzheimer disease in African Americans. *Alzheimers Dement* 2014; 10:618.e11.
23. Farrer LA, Cupples LA, Haines JL, *et al*. Effects of age, sex, and ethnicity on the association between apolipoprotein E genotype and Alzheimer disease. A meta-analysis. APOE and Alzheimer Disease Meta Analysis Consortium. *JAMA* 1997; 278:1349-1356.
24. Corder EH, Saunders AM, Strittmatter WJ, *et al*. Gene dose of apolipoprotein E type 4 allele and the risk of Alzheimer's disease in late onset families. *Science* 1993; 261:921-923.
25. Goate A, Chartier-Harlin MC, Mullan M, *et al*. Segregation of a missense mutation in the amyloid precursor protein gene with familial Alzheimer's disease. *Nature* 1991; 349:704-706.
26. Seshadri S, Fitzpatrick AL, Ikram MA, *et al*. Genome-wide analysis of genetic loci associated with Alzheimer disease. *JAMA* 2010; 303:1832-1840.
27. Jun GR, Chung J, Mez J, Barber R, *et al*. Transethnic genome-wide scan identifies novel Alzheimer's disease loci. *Alzheimers Dement* 2017; 13 727-738.
28. Rosenthal SL, Barmada MM, Wang X, Demirci FY, Kamboh MI. Connecting the dots: potential of data integration to identify regulatory SNPs in late-onset Alzheimer's disease GWAS findings. *PLoS ONE* 2014; 9:e95152.

© 2019 Patel D et al. *JAMA Network Open.*

29. Rehker J, Rodhe J, Nesbitt RR, *et al*. Caspase-8, association with Alzheimer's Disease and functional analysis of rare variants. *PLoS ONE* 2017; 12:e0185777.

30. Lambert JC, Ibrahim-Verbaas CA, Harold D, *et al*. Meta-analysis of 74,046 individuals identifies 11 new susceptibility loci for Alzheimer's disease. *Nat Genet* 2013; 45:1452-1458.

31. Bannwarth S, Ait-El-Mkadem S, Chaussenot A, *et al*. A mitochondrial origin for frontotemporal dementia and amyotrophic lateral sclerosis through CHCHD10 involvement. *Brain* 2014; 137(Pt 8):2329-2345.

32. Skibinski G, Parkinson NJ, Brown JM, *et al*. Mutations in the endosomal ESCRTIII-complex subunit CHMP2B in frontotemporal dementia. *Nat Genet* 2005; 37:806-808.

33. Mez J, Chung J, Jun G, *et al*. Two novel loci, *COBL* and *SLC10A2*, for Alzheimer's disease in African Americans. *Alzheimers Dement* 2017; 13 119-129.

34. Rademakers R, Baker M, Nicholson AM, *et al*. Mutations in the colony stimulating factor 1 receptor (CSF1R) gene cause hereditary diffuse leukoencephalopathy with spheroids. *Nat Genet* 2011; 44:200-205.

35. van der Knaap, Marjo S., Leegwater PAJ, *et al*. Mutations in each of the five subunits of translation initiation factor eIF2B can cause leukoencephalopathy with vanishing white matter. *Ann Neurol* 2002; 51:264-270.

36. Matsukawa T, Wang X, Liu R, Wortham NC, Onuki Y, Kubota A, et al. Adult-onset leukoencephalopathies with vanishing white matter with novel missense mutations in EIF2B2, EIF2B3, and EIF2B5. Neurogenetics. 2011; 12(3):259-261.

37. Tosto G, Fu H, Vardarajan BN, *et al*. F-box/LRR-repeat protein 7 is genetically associated with Alzheimer's disease. *Ann Clin Transl Neurol* 2015; 2:810-820.

38. Lambert J-, Grenier-Boley B, Harold D, *et al*. Genome-wide haplotype association study identifies the FRMD4A gene as a risk locus for Alzheimer's disease. *Mol Psychiatry* 2013; 18:461-470.

39. Yan J, Deng H, Siddique N, *et al*. Frameshift and novel mutations in FUS in familial amyotrophic lateral sclerosis and ALS/dementia. *Neurology* 2010; 75:807-814.

40. Cruchaga C, Kauwe JSK, Harari O, *et al*. GWAS of cerebrospinal fluid tau levels identifies risk variants for Alzheimer's disease. *Neuron* 2013; 78:256-268.

41. Rademakers R, Eriksen JL, Baker M, *et al*. Common variation in the miR-659 binding-site of GRN is a major risk factor for TDP43-positive frontotemporal dementia. *Hum Mol Genet* 2008; 17:3631-3642.

42. Lee JH, Kahn A, Cheng R, *et al*. Disease-related mutations among Caribbean Hispanics with familial dementia. *Mol Genet Genomic Med* 2014; 2:430-437.

43. Wang Z, Wan Y, Tan L, *et al*. Genetic Association of HLA Gene Variants with MRI Brain Structure in Alzheimer's Disease. *Mol Neurobiol* 2017; 54:3195-3204.

44. Escott-Price V, Bellenguez C, Wang L, *et al*. Gene-wide analysis detects two new susceptibility genes for Alzheimer's disease. *PLoS ONE* 2014; 9:e94661.

45. Vidal R, Frangione B, Rostagno A, *et al*. A stop-codon mutation in the BRI gene associated with familial British dementia. *Nature* 1999; 399:776-781.

46. Ghiso JA, Holton J, Miravalle L, *et al*. Systemic amyloid deposits in familial British dementia. *J Biol Chem* 2001; 276:43909-43914.

47. Vidal R, Revesz T, Rostagno A, *et al*. A decamer duplication in the 3' region of the BRI gene originates an amyloid peptide that is associated with dementia in a Danish kindred. *Proc Natl Acad Sci USA* 2000; 97:4920-4925.

48. Jun G, Ibrahim-Verbaas CA, Vronskaya M, *et al*. A novel Alzheimer disease locus located near the gene encoding tau protein. *Mol Psychiatry* 2016; 21:108-117.

49. Schuster J, Sundblom J, Thuresson A, *et al*. Genomic duplications mediate overexpression of lamin B1 in adult-onset autosomal dominant leukodystrophy (ADLD) with autonomic symptoms. *Neurogenetics* 2011; 12:65-72.

50. Beecham G, Hamilton K, Naj A, *et al*. Genome-wide association meta-analysis of neuropathologic features of Alzheimer's disease and related dementias. *PLoS Genet* 2014; 10:e10

51. Chung J, Wang X, Maruyama T, *et al*. Genome-wide association study of Alzheimer's disease endophenotypes at prediagnosis stages. *Alzheimers Dement* 2018; 14:623-633.

52. McKenzie AT, Moyon S, Wang M, *et al*. Multiscale network modeling of oligodendrocytes reveals molecular components of myelin dysregulation in Alzheimer's disease. *Mol Neurodegener* 2017; 12:82.

53. Joutel A, Corpechot C, Ducros A, *et al*. Notch3 mutations in CADASIL, a hereditary adult-onset condition causing stroke and dementia. *Nature* 1996; 383:707-710.

54. Joutel A, Vahedi K, Corpechot C, *et al*. Strong clustering and stereotyped nature of Notch3 mutations in CADASIL patients. *Lancet* 1997; 350:1511-1515.

55. Patel T, Brookes KJ, Turton J, *et al*. Whole-exome sequencing of the BDR cohort: evidence to support the role of the PILRA gene in Alzheimer's disease. *Neuropathol Appl Neurobiol* 2017. doi:10.1111/nan.12452.

56. Logue MW, Schu M, Vardarajan BN, *et al*. Search for age-related macular degeneration risk variants in Alzheimer disease genes and pathways. *Neurobiol Aging* 2014; 35(6):18.

© 2019 Patel D et al. *JAMA Network Open.*

57. Cruchaga C, Karch CM, Jin SC, *et al*. Rare coding variants in the phospholipase D3 gene confer risk for Alzheimer's disease. *Nature* 2014; 505:550-554.

58. Jun G, Asai H, Zeldich E, *et al*. PLXNA4 is associated with Alzheimer disease and modulates tau phosphorylation. *Ann Neurol* 2014; 76:379-392.

59. Jun GR, Chung J, Lunetta KL, *et al*. Further stratification of APOE E4-negative subjects identifies novel genes for Alzheimer's disease. *Alzheimers Dement* 2016; 12(7).

60. Mead S, Mahal SP, Beck J, *et al*. Sporadic--but not variant--Creutzfeldt-Jakob disease is associated with polymorphisms upstream of PRNP exon 1. *Am J Hum Genet* 2001; 69:1225-1235.

61. Sherrington R, Rogaev EI, Liang Y, *et al*. Cloning of a gene bearing missense mutations in early-onset familial Alzheimer's disease. *Nature* 1995; 375:754-760.

62. Levy-Lahad E, Wijsman EM, Nemens E, *et al*. A familial Alzheimer's disease locus on chromosome 1. *Science* 1995; 269:970-973

63. Xu W, Fang F, Ding J, Wu C. Dysregulation of Rab5-mediated endocytic pathways in Alzheimer's disease. *Traffic* 2018; 19:253-262.

64. Reitz C, Tokuhiro S, Clark LN, *et al*. SORCS1 alters amyloid precursor protein processing and variants may increase Alzheimer's disease risk. *Ann Neurol* 2011; 69:47-64.

65. Reitz C, Tosto G, Vardarajan B, *et al*. Independent and epistatic effects of variants in VPS10-d receptors on Alzheimer disease risk and processing of the amyloid precursor protein (APP). *Transl Psychiatry* 2013; 3:e256.

66. Rogaeva E, Meng Y, Lee JH, *et al*. The neuronal sortilin-related receptor SORL1 is genetically associated with Alzheimer’s disease. *Nat Genet* 2007; 39:168-177.

67. Kovacs GG, Murrell JR, Horvath S, *et al*. TARDBP variation associated with frontotemporal dementia, supranuclear gaze palsy, and chorea. *Mov Disord* 2009; 24:1843-1847.

68. Jakobsdottir J, van der Lee SJ, Bis JC, *et al*. Rare functional variant in TM2D3 is associated with late-onset Alzheimer's disease. *PLoS Genet* 2016; 12:e1006456.

69. Jonsson T, Stefansson H, Steinberg S, *et al*. Variant of TREM2 associated with the risk of Alzheimer's disease. *N Engl J Med* 2013; 368:107-116.

70. Guerreiro R, Wojtas A, Bras J, *et al*. TREM2 variants in Alzheimer's disease. *N Engl J Med* 2013; 368:117-127.

71. Ruiz A, Heilmann S, Becker T, *et al*. Follow-up of loci from the International Genomics of Alzheimer's Disease Project identifies TRIP4 as a novel susceptibility gene. *Transl Psychiatry* 2014; 4:e358.

72. Wetzel-Smith MK, Hunkapiller J, Bhangale TR, *et al*. A rare mutation in UNC5C predisposes to late-onset Alzheimer's disease and increases neuronal cell death. *Nat Med* 2014; 20:1452-1457.

73. Mehta SG, Khare M, Ramani R, *et al*. Genotype-phenotype studies of VCP-associated inclusion body myopathy with Paget disease of bone and/or frontotemporal dementia. *Clin Genet* 2013; 83:422-431.

74. Kauwe JSK, Jacquart S, Chakraverty S, et al. Extreme cerebrospinal fluid amyloid beta levels identify family with late-onset Alzheimer's disease presenilin 1 mutation. *Ann Neurol* 2007; 61:446-453.

75. Fernández MV, Black K, Carrell D, et al. SORL1 variants across Alzheimer's disease European American cohorts. *Eur J Hum Genet* 2016; 24:1828-1830.

76. Guerreiro RJ, Baquero M, Blesa R, et al. Genetic screening of Alzheimer's disease genes in Iberian and African samples yields novel mutations in presenilins and APP. *Neurobiol Aging* 2010; 31:725-731.

77. Lindquist SG, Holm IE, Schwartz M, et al. Alzheimer disease-like clinical phenotype in a family with FTDP-17 caused by a MAPT R406W mutation. *Eur J Neurol* 2008; 15:377-385.

78. Vardarajan BN, Ghani M, Kahn A, et al. Rare coding mutations identified by sequencing of Alzheimer disease genome-wide association studies loci. *Ann Neurol* 2015; 78:487-498.

79. Price AL, Patterson NJ, Plenge RM, Weinblatt ME, Shadick NA, Reich D. Principal components analysis corrects for stratification in genome-wide association studies. *Nat Genet* 2006; 38:904-909.

80. Patterson N, Price AL, Reich D. Population structure and eigenanalysis. *PLoS Genet* 2006; 2, e190.

81. Szklarczyk D, Morris JH, Cook H, et al. The STRING database in 2017: quality-controlled protein–protein association networks, made broadly accessible. *Nucleic Acids Res* 2017; 45:D368.

© 2019 Patel D et al. *JAMA Network Open.*

eTable 1. Characteristics of Subjects in the ADSP WES Case-Control Dataset

Group	N	Participants with AD	Controls	Males	Females	Mean Age (SD) *	% *APOE* ε4 carrier
European ancestry	10,441	5,617 (54%)	4,594 (44%)	4,411 (42%)	6,030 (58%)	81.0 (9.1)	30%
Caribbean Hispanic	395	218 (55%)	177 (45%)	149 (38%)	246 (62%)	74.4 (7.8)	40%

* Age at onset for AD cases, age at exam for controls

© 2019 Patel D et al. *JAMA Network Open.*

eTable 2. Previously Established Genes for AD, AD-Related Traits and Other Dementias

Gene	Disease or Trait	Reference	Gene	Disease or Trait	Reference
**ABCA7**	AD	[10,13,14]	**KCNMB2**	AD	[15]
**ABCG1**	NP	[15]	**LMNB1**	Leukodystrophy	[49]
**ABI3**	AD	[16]	**LMX1B**	HS	[50]
**ACE**	AD	[17]	**MAPT**	AD	[48]
**ADAM10**	AD	[18, 19]	**MEF2C**	AD	[30]
**ADAMTS1**	AD	[20]	**MS4A4A**	AD	[11]
**ADI1**	NFT + CAA	[21]	**MS4A6A**	AD	[30]
**AKAP9**	AD	[22]	**MTUS1**	HPV	[51]
**APOE**	AD	[23, 24]	**MVB12B**	AD	[52]
**APP**	AD	[25]	**NME8**	AD	[30]
**BIN1**	AD	[26]	**NOTCH3**	CADASIL	[53, 54]
**BZRAP1**	AD	[27]	**OSTN**	AD	[40]
**C1QTNF4**	AD	[28]	**PDGFRL**	HPV	[51]
**CASP8**	AD	[29]	**PFDN1**	AD	[27]
**CASS4**	AD	[30]	**PICALM**	AD	[30]
**CD2AP**	AD	[30]	**PILRA**	AD	[55, 56]
**CD33**	AD	[30]	**PLCG2**	AD	[16]
**CELF1**	AD	[30]	**PLD3**	AD	[57]
**CHCHD10**	FTD	[31]	**PLD4**	CSF Tau	[51]
**CHMP2B**	FTD	[32]	**PLXNA4**	AD	[58]
**CLU**	AD	[31]	**PPP2CB**	AD	[59]
**COBL**	AD	[33]	**PRNP**	CJD / GSS	[60]
**CR1**	AD	[30]	**PSEN1**	AD	[61]
**CSF1R**	FTD	[34]	**PSEN2**	AD	[62]
**DSG2**	AD	[30]	**PTK2B**	AD	[30]
**ECHDC3**	AD	[26]	**RIN3**	AD	[63]
**ECRG4**	NP + NFT	[21]	**SLC10A2**	AD	[33]
**EIF2B1**	Leukodystrophy	[35]	**SLC24A4**	AD	[30]
**EIF2B2**	Leukodystrophy	[36]	**SLC2A4A**	AD	[51]
**EIF2B3**	Leukodystrophy	[36]	**SORCS1**	AD	[64, 65]
**EIF2B4**	Leukodystrophy	[35]	**SORCS2**	AD	[65]
**EIF2B5**	Leukodystrophy	[36]	**SORCS3**	AD	[65]
**EPHA1**	AD	[10, 14]	**SORL1**	AD	[66]
**FBXL7**	AD	[37]	**SRRM4**	CSF Tau	[51]
**FERMT2**	AD	[30]	**TARDBP**	FTD	[67]
**FRMD4A**	AD	[38]	**TM2D3**	AD	[68]
**FUS**	FTD	[39]	**TP53INP1**	AD	[44]
**GALNT7**	NP	[15]	**TPBG**	AD	[27]
**GLIS3**	AD	[40]	**TRAPPC12**	NFT + CAA	[21]
**GRN**	FTD	[41, 42]	**TREM2**	AD	[69, 70]
**HBEGF**	AD	[27]	**TREML2**	AD	[40]
**HDAC9**	NFT + CAA	[21]	**TRIP4**	AD	[71]
**HLA-DRB1**	AD	[43]	**UNC5C**	AD	[72]
**HLA-DRB5**	AD	[30]	**USP6NL**	AD	[27]
**IGHV1-67**	AD	[44]	**VCP**	FTD	[73]
**INPP5D**	AD	[30]	**ZCWPW1**	AD	[30, 56]
**ITM2B**	FBD /FDD	[45-47]	**ZNF804B**	LMdT	[51]
**KANSL1**	AD	[48]			

© 2019 Patel D et al. *JAMA Network Open.*

AD = Alzheimer disease; CAA = cerebral amyloid angiopathy; CADASIL = Cerebral Autosomal Dominant Arteriopathy with Subcortical Infarcts and Leukoencephalopathy; CJD = Creutzfeldt-Jakob disease; FBD = Familial British dementia; FDD = Familial Danish dementia; FTD = fronto-temporal dementia; GSS = Gerstmann-Straussler syndrome; HPV = hippocampal volume; HS = hippocampal sclerosis; LMdT = logical memory – delayed recall; NFT = neurofibrillary tangle; NP = neuritic plaque

© 2019 Patel D et al. *JAMA Network Open.*

eTable 3. Filtering Pipeline of Rare Variants

**# of Variants (# of Genes)**	**Filtering Steps**
1,015,329 (29,157)	Total rare variants genome-wide after counts are calculated with PLINK
75,716 (15,702)	Total rare variants genome-wide after filtering (to remove synonymous mutations, those with a count less than or equal to 2) and restricted to those with a high or moderate impact on disease (as classified by VEP annotation).
4,854 (3,619)	Total rare variants genome-wide after filtering and with high impact on disease.
10,249 (95)	Total rare variants in known AD genes
503 (83)	Total rare variants in known AD genes after filtering and with high and moderate impact on disease.
24 (19)	Total rare variants in known AD genes after filtering and with high impact on disease.

© 2019 Patel D et al. *JAMA Network Open.*

eTable 4. High and Moderate Impact Rare Variants in Previously Established AD Genes Occurring In ≥ 4 Participants With AD and No Controls**.**

(Chromosome: position: major allele: minor allele) *	MAC	Gene	ID	Frequency in GnomAD	Mutation Type	Amino Acid Position and Change	Disease Impact	CADD Score	Previously Associated with AD
19:15302421:C:T	10	*NOTCH3*	rs149307620	T=0.00031	Missense	A284T	Moderate	24.7	No
14:73637653:C:T	7	*PSEN1*	rs63749824	T=0.00001	Missense	A75V	Moderate	27.1	[74]
9:4118361:G:C	6	*GLIS3*	rs200263979	C=0.00104	Missense	L373V	Moderate	15.67	No
14:93154397:G:A	6	*RIN3*	Novel	NA	Missense	NA	Moderate	18.65	No
17:44143925:C:T	6	*KANSL1*	rs138698439	T=0.00046	Missense	S609N	Moderate	20.2	No
11:121384991:A:G	5	*SORL1*	rs139710266	G=0.00003	Missense	Y391C	Moderate	25.8	[75]
14:73640432:G:A	5	*PSEN1*	rs375376095	A=0.00007	Missense	NA	Moderate	10.23	No
16:31193959:ATTC:A	5	*FUS*	Novel	NA	In-frame deletion	NA	Moderate	13.39	No
17:56385997:G:A	5	*BZRAP1*	rs61732758	A=0.00374	Missense	P1546S	Moderate	16.91	No
19:1054190:A:G	5	*ABCA7*	rs376824416	G=0.00010	Splice acceptor	NA	High	23.1	No
2:234113197:C:T	4	*INPP5D*	rs532718867	T=0.00036	Missense	P1133L	Moderate	0.44	No
4:7731386:G:A	4	*SORCS2*	rs371407070	A=0.00011	Missense	V1019L	Moderate	24	No
6:41129295:G:A	4	*TREM2*	rs104894002	A=0.00002	Stop Gained	Q33X	High	33	[70]
7:143088779:C:T	4	*EPHA1*	rs201365734	G=0.01583	Missense	A712P	Moderate	5.88	No
7:51096830:C:G	4	*COBL*	rs112568753	T=0.00025	Missense	R929H	Moderate	25.1	No
8:17478643:A:G	4	*PDGFRL*	rs144384825	G=0.00019	Missense	Q146R	Moderate	18.08	No
9:4118324:C:A	4	*GLIS3*	rs200959196	A=0.00021	Missense	G385V	Moderate	9.21	No
11:59949160:A:G	4	*MS4A6A*	Novel	NA	Missense	NA	Moderate	21.1	No
14:105396368:T:G	4	*PLD4*	Novel	A=0.00018	Missense	R35Q	Moderate	16.45	No
14:73637521:G:A	4	*PSEN1*	rs63750592	NA	Missense	R35Q	Moderate	22.1	[76]
14:93154353:T:TGTGCGCGCA	4	*RIN3*	Novel	NA	In-frame insertion	NA	Moderate	23.2	No
15:102192524:A:G	4	*TM2D3*	rs201415552	G=0.00005	Missense	L14S	Moderate	8.86	No
16:81972496:A:G	4	*PLCG2*	Novel	NA	Missense	NA	Moderate	22.6	No
17:42429772:C:T	4	*GRN*	Novel	NA	Stop Gained	NA	High	36	No
17:44101427:C:T	4	*MAPT*	rs63750424	T=0.00002	Missense	A741W	Moderate	29.8	[77]
17:61568688:G:A	4	*ACE*	rs143507892	A=0.00113	Missense	R953Q	Moderate	29.5	No
17:61574683:C:T	4	*ACE*	Novel	NA	Missense	NA	Moderate	11.91	No
19:1043794:G:A	4	*ABCA7*	rs147846250	A=0.00024	Missense	R334Q	Moderate	5.43	No
19:1055151:T:C	4	*ABCA7*	rs145987355	NA	Missense	NA	Moderate	26.5	No
19:1058154:G:T	4	*ABCA7*	rs770510230	T=0.00001	Stop Gained	E1679X	High	39	[78]
19:1059029:G:A	4	*ABCA7*	rs143615723	A=0.00015	Missense	R1803H	Moderate	0.73	No
19:15272218:G:A	4	*NOTCH3*	rs114447350	A=0.02775	Missense	P2074Q	Moderate	22.2	No

* Position according to GRCh38.p7 assembly; MAC = minor allele count; CADD = Combined Annotation Dependent Depletion; NA = not available

© 2019 Patel D et al. *JAMA Network Open.*

eTable 5. Characteristics of AD Subjects in the ADSP WES Dataset With the *NOTCH3* rs149307620 Mutation

Study (Dataset)	Sex	Age at Onset	*APOE* Genotype	Braak Stage
ADGC (ADC2)	F	71	33	NA
ADGC (ADC3)	M	76	33	6
ADGC (ADC3)	M	78	34	NA
ADGC (ADC4)	F	74	34	NA
ADGC (ADC5)	M	78	24	NA
ADGC (Memory and Aging Project)	M	84	33	NA
ADGC (Texas Alzheimer's Research and Care Consortium)	F	80	33	NA
CHARGE	M	79	33	NA
CHARGE	M	95	33	NA
CHARGE	F	84	33	NA

NA = Not autopsied

© 2019 Patel D et al. *JAMA Network Open.*

eTable 6. Characteristics of Subjects in the WGS Replication Datasets

Dataset	N	AD Cases	MCI Cases	Controls	% Female	Mean Age (SD)	% *APOE* ε4 carrier
European ancestry ADSP Extension	1097	485	0	612	63.5%	78.4 (7.4)	38.5%
Caribbean Hispanic ADSP Extension	1018	493	0	525	55.1%	73.9 (7.4)	29.4%
African American ADSP Extension	977	454	0	523	55.1%	79.2 (7.1)	43.3%
European ancestry ADSP families	499	320	0	179	64.5%	73.4 (10.6)	39.3%
Caribbean Hispanic ADSP families	290	200	0	90	62.8%	70.6 (10.1)	32.5%
African American ADSP families	44	30	0	14	65.3%	69.1 (12.1)	51.0%
ADNI	809	239	321	249	44.8%	76.3 (8.1)	44.0%
**Total**	**4,734**	**2,221**	**321**	**2,192**			

© 2019 Patel D et al. *JAMA Network Open.*

eTable 7. Characteristics of AD Subjects With the *TREM2* rs104894002 Mutation (Q33X)

Source (Study)	Sex	Onset Age	APOE Genotype	Braak Stage
ADGC (ADC)	M	75	23	5
ADGC (Mayo Clinic)	M	63.3	44	NA
National Cell Repository for Alzheimer's Disease	F	69	34	4
CHARGE (Rotterdam Study)	M	73.5	34	NA

NA = not autopsied

© 2019 Patel D et al. *JAMA Network Open.*

eTable 8. High and Moderate Impact Rare Variants Genome-Wide Occurring in > 10 Participants With AD and No Controls.

(Chromosome: position: major allele: minor allele) *	MAC	Gene	ID	Frequency in GnomAD	Mutation Type	Amino Acid Position and Change	Disease Impact	CADD Score
11:5474894:T:C	12	*OR51I2*	rs74049540	C=0.01256	Missense	M59T	Moderate	24
19:38742032:G:A	12	*PPP1R14A*	rs140507040	A=0.00062	Missense	L97F	Moderate	16.24
14:45374714:CTTG:C	11	*C14orf28*	Novel	NA	In-frame deletion	NA	Moderate	20.1
17:42433931:C:G	11	*FAM171A2*	rs190723348	A=0.00023	Missense	L88	Moderate	19.26
17:7164266:G:A	11	*CLDN7*	rs149308129	G=0.00056	Missense	Q155H	Moderate	23.4
20:62198583:G:A	11	*HELZ2*	rs35691275	A=0.00053	Missense	R141W	Moderate	16.17
22:46759981:G:C	11	*CELSR1*	rs61741871	C=0.00766	Missense	P2983A	Moderate	3.6
22:46762988:C:T	11	*CELSR1*	rs75983687	T=0.00741	Missense	V2703M	Moderate	17
1:93682193:A:T	10	*CCDC18*	rs191574433	T=0.00034	Missense	M573L	Moderate	17.01
7:150389837:TC:T	10	*GIMAP2*	Novel	NA	Frameshift	NA	High	22.8
9:33941796:C:T	10	*UBAP2*	rs150194348	T=0.00078	Missense	V594I	Moderate	21.2
11:67957408:C:T	10	*SUV420H1*	rs138431226	T=0.00036	Missense	A46T	Moderate	15.32
12:88456506:T:C	10	*CEP290*	Novel	NA	Missense	NA	Moderate	25.3
14:31119819:C:A	10	*SCFD1*	rs35187633	A=0.00029	Missense	L173I	Moderate	27.2
17:74288944:A:G	10	*QRICH2*	Novel	A=0.01587	Missense	G248=	Moderate	15.81
14:74754936:G:A	10	*ABCD4*	rs57773157	T=0.01605	Missense	V172I	Moderate	4.738
14:74763064:C:T	10	*ABCD4*	rs34992370	C=0.01630	Missense	Q59R	Moderate	15.35
14:74766360:T:C	10	*ABCD4*	rs58272575	C=0.00217	Missense	D1330E	Moderate	15.68
17:58260659:A:C	10	*USP32*	rs201933998	NA	Missense	NA	Moderate	12.92
19:15302421:C:T	10	*NOTCH3*	rs149307620	T=0.00031	Missense	A284T	Moderate	24.7
19:49123796:G:A	10	*SPHK2*	Rs158184205	NA	Missense	NA	Moderate	16.44
22:41605776:G:C	10	*L3MBTL2*	rs143455680	C=0.01316	Missense	R34P	Moderate	27.4
22:46704734:C:T	10	*GTSE1*	rs34404175	T=0.00608	Missense	A219V	Moderate	5.9
22:46708152:G:A	10	*GTSE1*	rs35503220	A=0.00609	Missense	A293S	Moderate	0.001

*Position according to GRCh38.p7 assembly; MAC = minor allele count; CADD = Combined Annotation Dependent Depletion

© 2019 Patel D et al. *JAMA Network Open.*

eTable 9. Genes With ≥ 3 Distinct High/Moderate Disease Impact Rare Variants Each With a MAC ≥ 5 and Occurring in Only Cases

(Chromosome: position: major allele: minor allele) *	MAC	Gene	ID	Frequency in GnomAD	Mutation Type	Amino Acid Position and Change	Disease Impact	CADD Score
14:74754936:G:A	10	ABCD4	rs57773157	A=0.01587	Missense	G248=	Moderate	15.81
14:74766360:T:C	10	ABCD4	rs58272575	C=0.01630	Missense	Q59R	Moderate	4.738
14:74763064:C:T	10	ABCD4	rs34992370	T=0.01605	Missense	Va72I	Moderate	15.35
14:74764675:G:A	8	ABCD4	rs61744947	A=0.01600	Missense	A128V	Moderate	22.2
3:38125659:C:T	6	DLEC1	rs143610524	T=0.00078	Missense	A395V	Moderate	16.39
3:38101245:C:T	6	DLEC1	rs34012183	T=0.00326	Missense	S192F	Moderate	15.95
3:38101313:G:A	5	DLEC1	rs149190717	A=0.00100	Missense	D215N	Moderate	15.76
1:225239279:G:A	6	DNAH14	Novel	NA	Missense	NA	Moderate	12.93
1:225512438:G:A	5	DNAH14	rs566891789	A=0.00009	Splice Acceptor	NA	High	32
1:225458497:C:T	5	DNAH14	rs530417418	NA	Missense	NA	Moderate	28.1
7:111430638:G:A	5	DOCK4	rs377187510	A=0.00007	Missense	P1064S	Moderate	27.9
7:111617307:G:A	5	DOCK4	rs201242965	A=0.00016	Missense	P194L	Moderate	20.8
7:111503433:T:C	5	DOCK4	Novel	NA	Missense	NA	Moderate	22.5
9:133932442:C:T	7	LAMC3	rs113443891	T=0.00057	Missense	P689L	Moderate	19.73
9:133945155:G:A	7	LAMC3	rs113785045	A=0.00032	Missense	R996H	Moderate	22.3
9:133936490:A:G	6	LAMC3	rs36030184	G=0.00033	Missense	N743D	Moderate	17.38
9:133948124:G:A	5	LAMC3	rs144118534	A=0.00027	Missense	G1107R	Moderate	0.34
14:64898328:A:G	6	MTHFD1	rs139264994	G=0.00012	Missense	I464V	Moderate	18.17
14:64884726:C:T	6	MTHFD1	rs199501976	T=0.00024	Missense	A200V	Moderate	22.5
14:64916188:C:T	6	MTHFD1	rs17857382	T=0.00727	Missense	L769F	Moderate	20.5
1:228526693:C:T	5	OBSCN	rs370778898	T=0.00012	Missense	R5742C	Moderate	2.48
1:228558959:G:A	5	OBSCN	rs191931829	A=0.00026	Missense	R7784Q	Moderate	24.9
1:228437675:C:T	5	OBSCN	rs199655077	T=0.00016	Missense	A1348V	Moderate	25
14:88904567:G:A	6	SPATA7	rs10139784	A=0.01203	Missense	R534L	Moderate	9.96
14:88892697:G:A	6	SPATA7	rs17124662	A=0.00905	Missense	S165I	Moderate	0.74
14:88883100:A:G	6	SPATA7	rs61747004	G=0.00906	Missense	Q63R	Moderate	1.02
14:88895750:G:A	6	SPATA7	rs17124677	A=0.00905	Missense	G292E	Moderate	12.42
14:88883173:T:G	5	SPATA7	rs35137272	G=0.00908	Missense	F87L	Moderate	3.02
1:1269488:G:A	6	TAS1R3	rs112507608	A=0.01353	Missense	A735T	Moderate	10.39
1:1268954:C:T	5	TAS1R3	rs200679891	T=0.00018	Missense	R557C	Moderate	14.79

© 2019 Patel D et al. *JAMA Network Open.*

1:1268661:G:A	5	TAS1R3	rs373494410	A=0.00015	Missense	R501Q	Moderate	23.4
2:179499179:A:G	9	TTN	rs34706299	G=0.00033	Missense	V5045A	Moderate	23.4
2:179412829:C:T	6	TTN	rs72648251	T=0.00047	Missense	R28607H	Moderate	20.3
2:179452061:C:T	5	TTN	rs199505416	T=0.00008	Missense	D18725N	Moderate	23
2:179457733:G:A	5	TTN	rs72646839	A=0.00024	Missense	R10640C	Moderate	24.4
2:179440609:A:G	5	TTN	rs201836227	G=0.00022	Missense	I20849T	Moderate	24.5
2:179613715:C:G	5	TTN	rs145919543	G=0.00011	Missense	G4471A	Moderate	9.30

* Position according to GRCh38.p7 assembly; MAC = minor allele count; CADD = Combined Annotation Dependent Depletion; NA = not available

© 2019 Patel D et al. *JAMA Network Open.*

eTable 10. High Impact Rare Variants Genome-Wide With a MAC ≥ 7 and Occurring Only in AD Cases.

(Chromosome: position: major allele: minor allele) *	MAC	Gene	ID	Frequency in GnomAD	Mutation Type	Amino Acid Position and Change	Disease Impact	CADD Score
7:150389837:TC:T	10	GIMAP2	Novel	NA	Frameshift	NA	High	5.92
19:39926486:A:G	8	RPS16	rs139109626	G=0.00021	Splice donor	NA	High	22.4
11:5758174:CAATT:C	8	OR56B1	Novel	NA	Frameshift	NA	High	14.8
10:16979600:TGGTA:T	7	CUBN	rs556462218	NA	Frameshift	I1973V	High	40.0
10:25313667:T:A	7	THNSL1	rs150653385	A=0.00015	Stop gained	C505X	High	37.0
1:63063592:GAACTC:G	7	ANGPTL3	Novel	NA	Frameshift	NA	High	21.0
17:72350672:C:T	7	KIF19	rs200837156	T=0.00021	Stop gained	R894X	High	20.6
1:21904139:TG:T	7	ALPL	Novel	NA	Frameshift stop lost	NA	High	12.9
2:111918994:A:G	7	BCL2L11	rs76245002	G=0.00437	Splice acceptor	NA	High	5.26

* Position according to GRCh38.p7 assembly; MAC = minor allele count; CADD = Combined Annotation Dependent Depletion; NA = not available

© 2019 Patel D et al. *JAMA Network Open.*

eFigure 1. Study Design

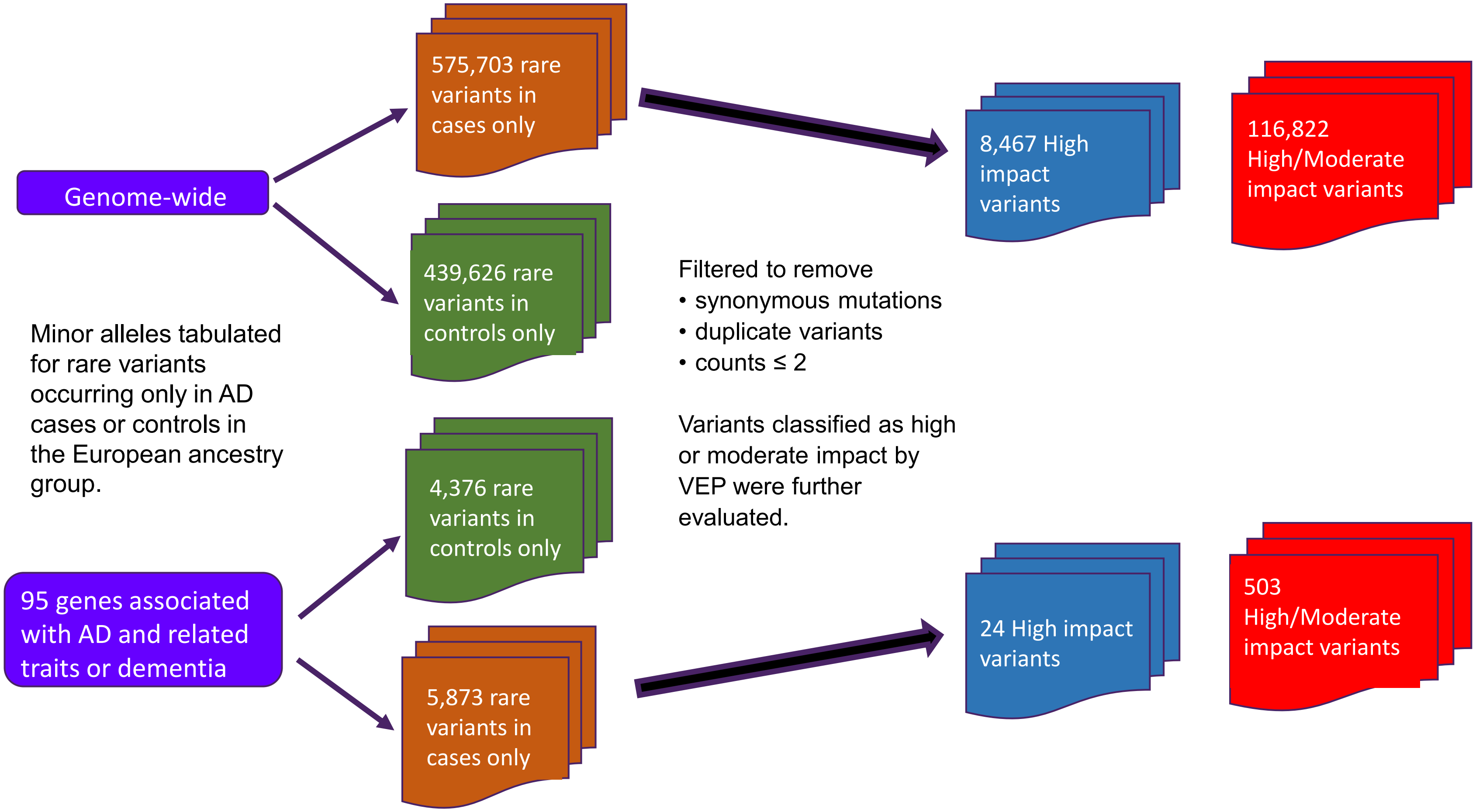

© 2019 Patel D et al. *JAMA Network Open.*

eFigure 2. Haplotype Analysis of the Rare *NOTCH3* rs149307620 Variant. All 10 subjects who have the rs149307620 mutation (marker 269) possess the GCCGC haplotype derived from five common SNPs (rs1548555, rs1044006, rs1043997, rs1043996, and rs1043994) spanning a region of ~24.8 kb. The haplotype has a frequency of 15.4% in AD cases and 14.7% in controls. Haplotypes and their corresponding frequencies are shown in the left panel. A measure of linkage disequilibrium (D') between each pair of SNPs is shown in the right panel.

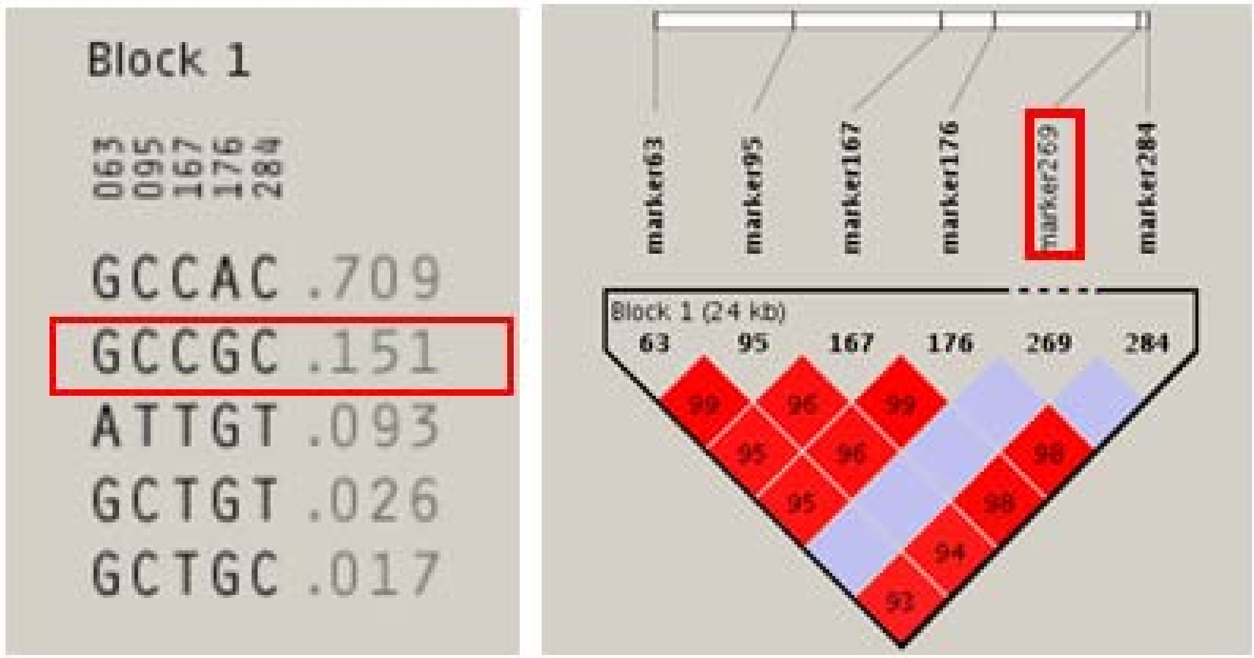

© 2019 Patel D et al. *JAMA Network Open.*

eFigure 3. Population Substructure of the ADSP Discovery Sample. Population substructure in the non-Hispanic European ancestry portion of the ADSP sample was evaluated by principal components (PC) analysis implemented in EIGENSTRAT[79,80] using the smartpca program as described previously.[2] For this analysis, variants were selected based on the following filtering criteria: MAF≥5%, call rate≥99%, and only one from each pair of variants with linkage disequilibrium (LD) of r^2>0.5 in a 50-variant window. Participants with individual call rate<90% were excluded, and only one participant from every pair with estimated $\hat{\pi}$>0.2 was selected. PCs were computed using the 1000 Genomes Phase 3 reference panel[6] and a subset of 12,351 variants that met the above criteria and were found in both ADSP and 1000G subjects. PCs were computed using all unrelated ADSP and 1000G subjects and projected on individuals that were omitted from the PC computation due to high IBD or low call rate. The plot of the first two PCs (PCA1 and PCA2) shows that participants (each represented by a small dot) are distributed along a population substructure gradient. A large portion of the persons in the cluster on the right side of the plot (PC1>0.025) have a mitochondrial haplogroup (K1a1b1a = light blue dots, K1a9 = dark blue dots) that is common among Ashkenazi Jews suggesting that this cluster includes primarily Ashkenazi Jews. Eight of the ten AD participants who have the *NOTCH3* rs149307620 mutation, including four persons with the K1a1b1a or K1a9 haplogroup (dark red + symbol) and four persons lacking either Ashkenazi haplogroup (brown + symbol), are included in this cluster.

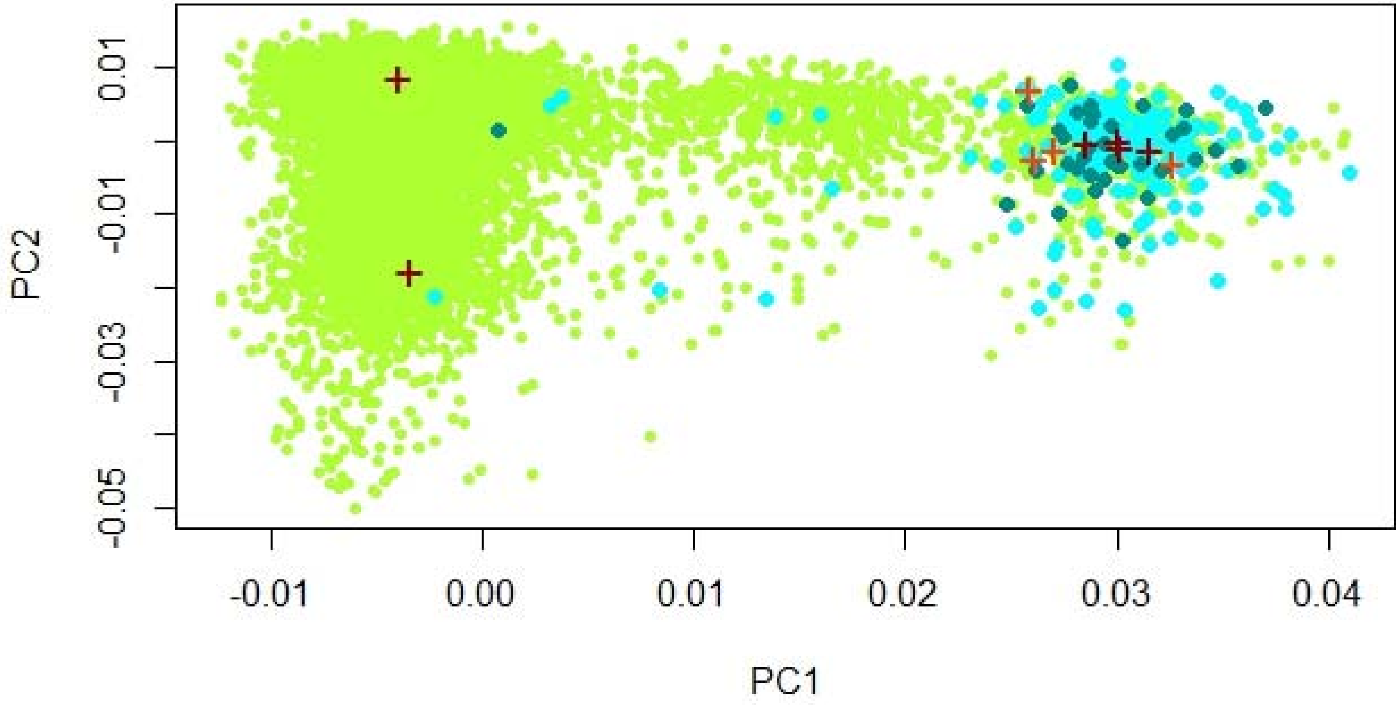

© 2019 Patel D et al. *JAMA Network Open.*

eFigure 4. Utah Pedigree Segregating rs141402160 and rs140914494 Mutations. Full shading indicates AD diagnosis from death certificate or hospital data; half shading indicates dementia diagnosis from death certificate or hospital data. For confidentiality purposes, alive/death status is not indicated and age shown is either + or – 5 years of the actual age or age at death or age >90. "+" indicates the presence of the minor allele at both rs141402160 and rs140914494, "-" indicates wild type at both variants, and "*" indicates that carrier status was inferred as positive do to the rarity of the variants and their presence in that individual's child (child not pictured in the pedigree due to lack of disease status information).

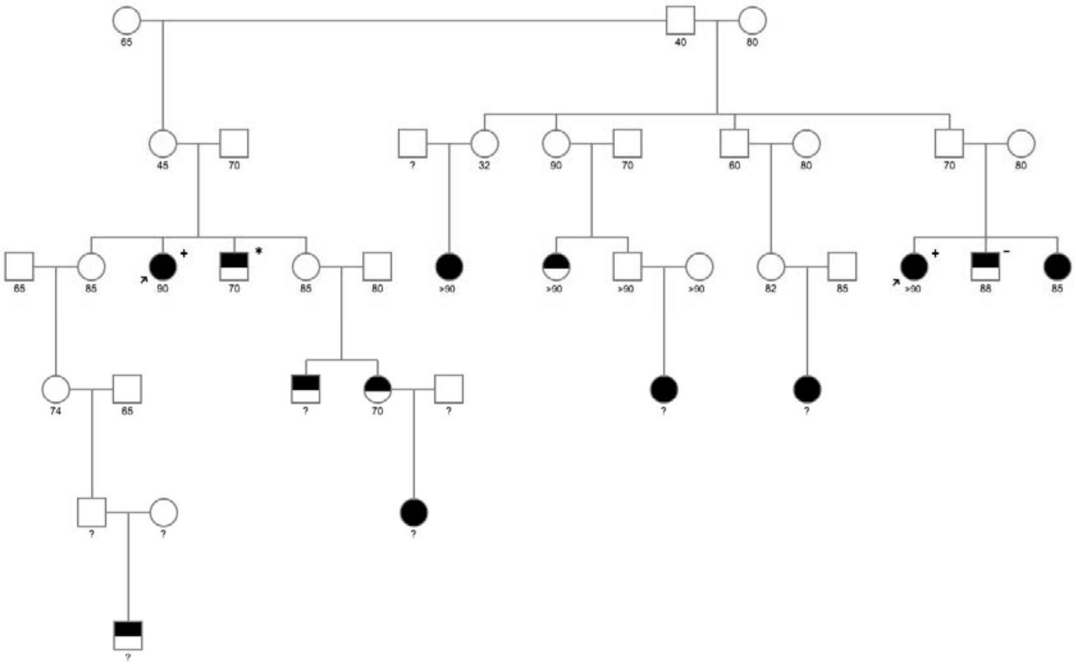

© 2019 Patel D et al. *JAMA Network Open.*

eFigure 5. Utah Pedigree Segregating rs112197217 Mutation. Full shading indicates AD diagnosis from death certificate or hospital data; half shading indicates dementia diagnosis from death certificate or hospital data. For confidentiality purposes, alive/death status is not indicated and age shown is either + or – 5 years of the actual age or age at death or age >90. “+” indicates that the individual is a carrier of the minor allele at rs112197217.

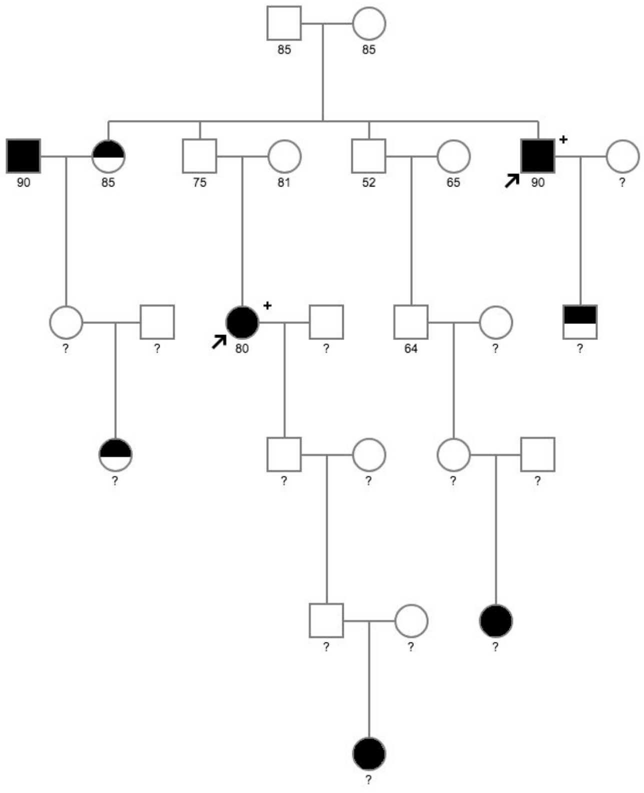

© 2019 Patel D et al. *JAMA Network Open.*

eFigure 6. Protein-Protein Interaction Network Including *NOTCH3* and *JAG1*. Network includes 30 genes showing high-confidence interactions in humans according to the STRING database.[81] The colors of the edges refer to the type of evidence linking the corresponding proteins: red=gene fusion, dark blue=co-occurrence, black=co-expression, magenta=experiments, cyan=databases, light green=text mining, mauve=homology. Genes present in AD pathways determined by gene-set enrichment analysis (Table2) are highlighted by yellow circles.

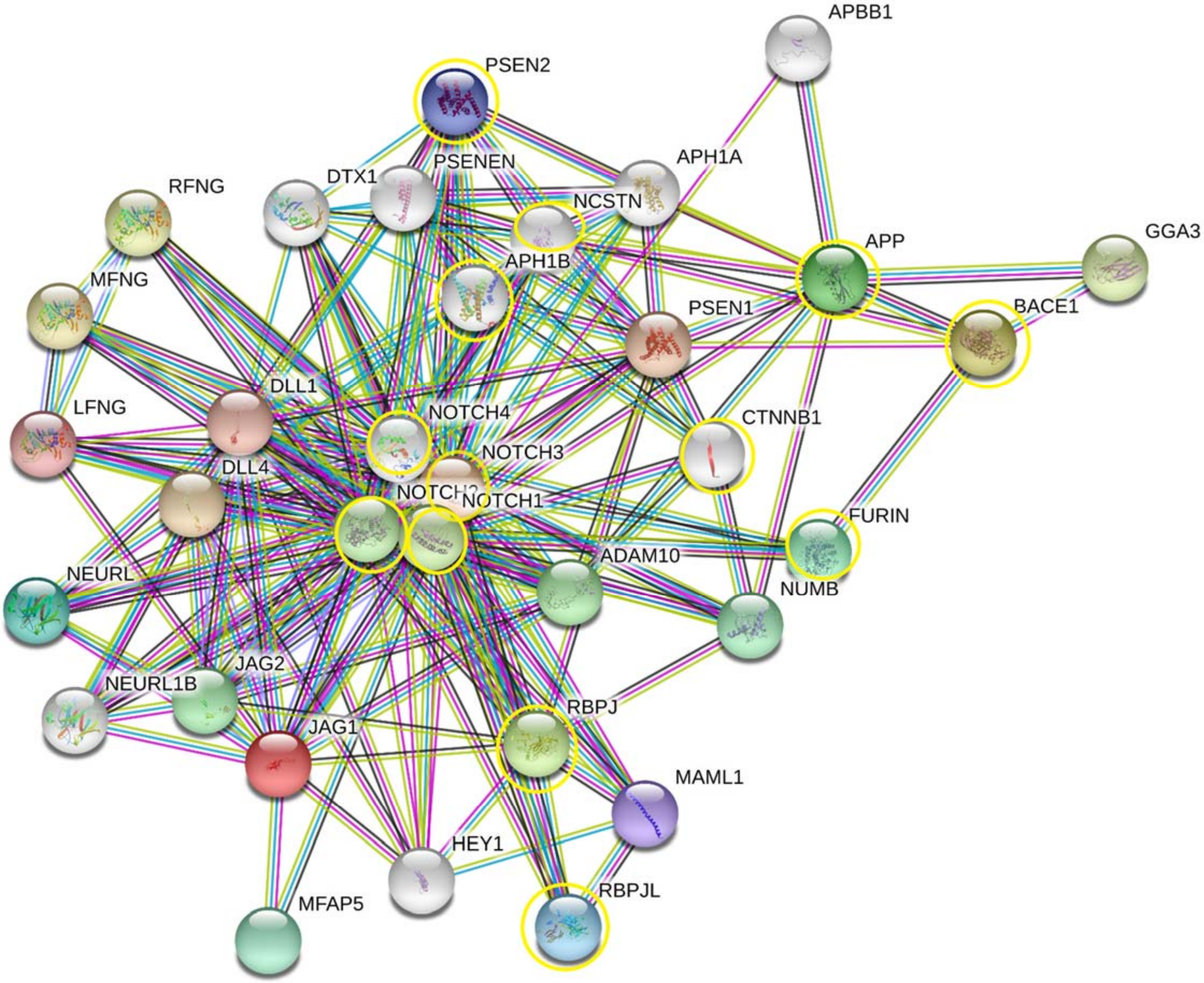

© 2019 Patel D et al. *JAMA Network Open.*

eFigure 7. Haplotype Analysis of *ABCD4* and *CELSR1/GTSE1*. **A.** All 10 subjects with ABCD4 rare variants rs57773157 (marker 55), rs34992370 (marker 130), and rs58272575 (marker 158) share an 8-SNP haplotype spanning ~12.9 kb that has a frequency of 0.3% in AD cases and 0% in controls. Eight of these subjects also have a fourth *ABCD4* rare variant, rs61744947 (marker148) that is part of the same shared haplotype. **B.** All eight subjects with *CELSR1* variants rs61741871 (marker 268) and rs75983687 (marker345) and *GTSE1* variants rs34404175 (marker 74) and rs35503220 (marker84) share a 12 SNP-haplotype spanning ~77.6 kb that has a frequency of 0.1% in the ADSP population (0.1% in cases, 0.1% in controls). Haplotypes and their corresponding frequencies are shown on the left side of each panel. A measure of linkage disequilibrium (D') between each pair of SNPs is shown on the right side of each panel.

A.

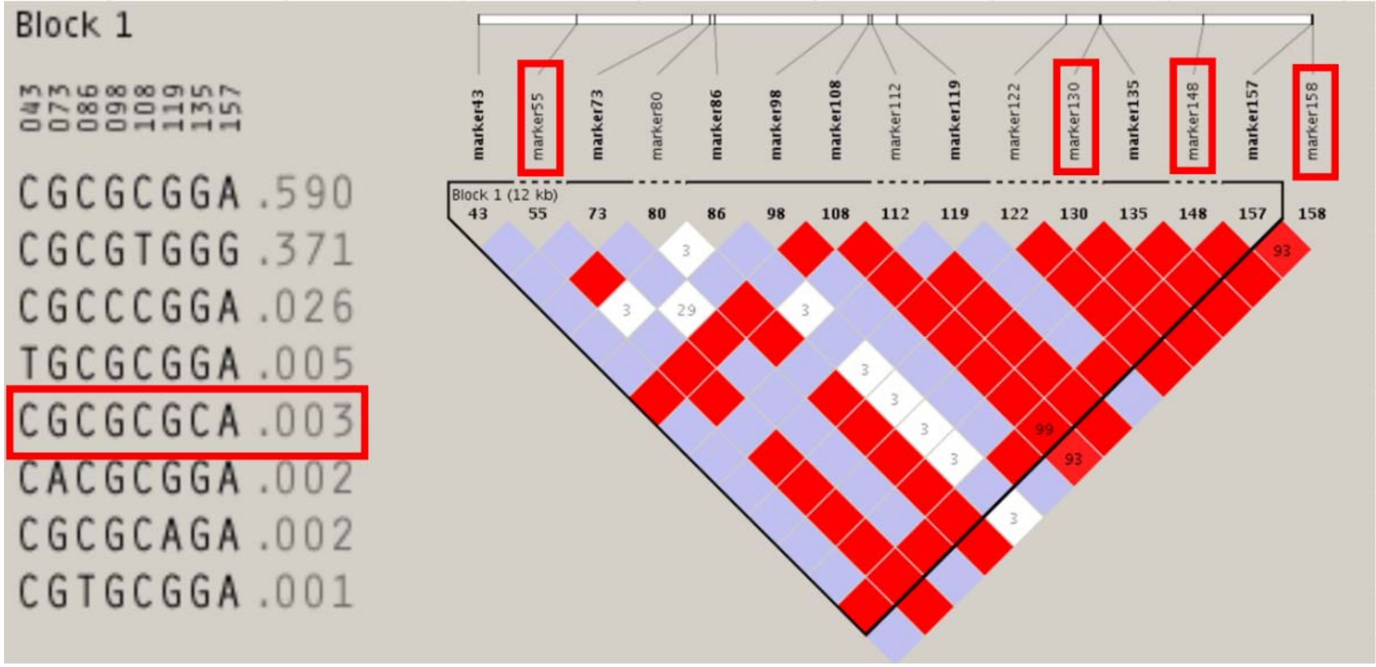

© 2019 Patel D et al. *JAMA Network Open.*

B.

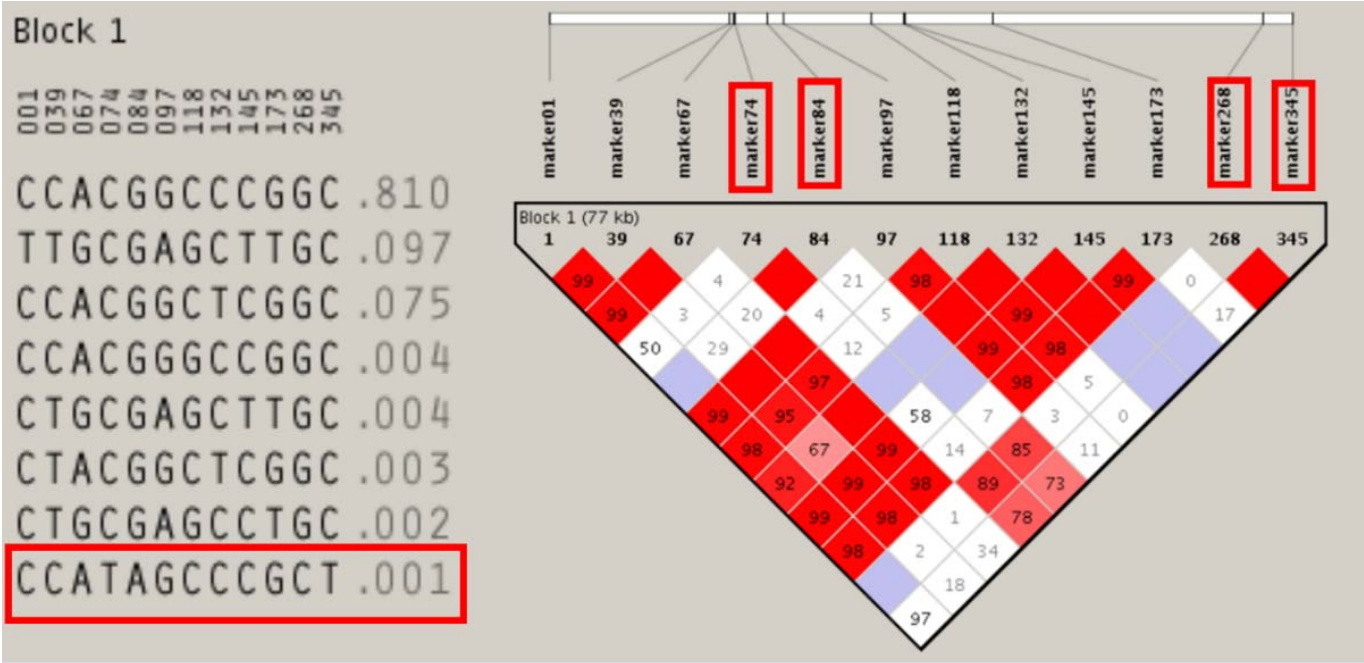

© 2019 Patel D et al. *JAMA Network Open.*
